# A stress-responsive enhancer induces dynamic drug resistance in acute myeloid leukemia

**DOI:** 10.1172/JCI130809

**Published:** 2020-02-04

**Authors:** Mark S. Williams, Fabio M.R. Amaral, Fabrizio Simeoni, Tim C.P. Somervaille

**Affiliations:** Leukaemia Biology Laboratory, Cancer Research UK Manchester Institute, University of Manchester, Manchester, United Kingdom.

**Keywords:** Hematology, Oncology, Cell stress, Leukemias

## Abstract

The drug efflux pump ABCB1 is a key driver of chemoresistance, and high expression predicts treatment failure in acute myeloid leukemia (AML). In this study, we identified and functionally validated the network of enhancers that controls expression of *ABCB1*. We show that exposure of leukemia cells to daunorubicin activated an integrated stress response–like transcriptional program to induce *ABCB1* through remodeling and activation of an ATF4-bound, stress-responsive enhancer. Protracted stress primed enhancers for rapid increases in activity following re-exposure of cells to daunorubicin, providing an epigenetic memory of prior drug treatment. In primary human AML, exposure of fresh blast cells to daunorubicin activated the stress-responsive enhancer and led to dose-dependent induction of *ABCB1*. Dynamic induction of *ABCB1* by diverse stressors, including chemotherapy, facilitated escape of leukemia cells from targeted third-generation ABCB1 inhibition, providing an explanation for the failure of ABCB1 inhibitors in clinical trials. Stress-induced upregulation of *ABCB1* was mitigated by combined use of the pharmacologic inhibitors U0126 and ISRIB, which inhibit stress signaling and have potential for use as adjuvants to enhance the activity of ABCB1 inhibitors.

## Introduction

Resistance of leukemia cells, including leukemia stem cells with disease-reconstituting activity, to the chemotherapy drugs used in standard induction and consolidation regimens is the most common cause of treatment failure in acute myeloid leukemia (AML). Primary drug resistance is certainly linked to the genetic lesions driving AML; for example, leukemias with an NPM1^mut^ FLT3-ITD genotype are substantially more difficult to cure with chemotherapy alone than NPM1^mut^ AMLs that lack FLT3-ITD, although the reasons for such differential sensitivity remain obscure. Levels of expression of drug-detoxifying enzymes, topoisomerase II, microRNAs, and the propensity of cells to undergo autophagy have all been suggested to contribute to intrinsic drug resistance (1).

Most significantly, high expression of the ABCB1 drug efflux pump (also known as MDR1 or P-glycoprotein), which actively exports anthracyclines, predicts treatment failure in AML (2, 3). More generally, ABCB1 is highly expressed in many poor-risk malignancies as well as in normal gut, liver, and kidney and the blood-brain barrier (4). Inhibitors of ABCB1 have been tested in clinical trials in AML but with limited success. Nevertheless, in view of its significant role in the disease, the rationale for targeting ABCB1 remains strong (3). Furthermore, given the abundance of preclinical evidence supporting a role for ABCB1 in drug resistance, the failure in clinical trials of inhibitors of ABCB1 has not been adequately explained.

A greater understanding of the cancer-specific regulation of ABCB1 and its role in drug resistance is required to facilitate the design of new therapeutic strategies. Specifically, it is unclear how *ABCB1* expression is established and maintained in human AML. Whether expression is constitutive or dynamic is of critical relevance to the clinical application of ABCB1 inhibitors, where previous trials have assumed constant expression (5). Advances in enhancer biology have established that these distal regulatory elements govern cell type–specific gene expression and frequently respond to environmental conditions and homeostatic perturbations (6, 7). Critically, the enhancer landscape of *ABCB1* has yet to be defined.

## Results

### Resistance to daunorubicin due to stereotypical induction of ABCB1.

We initially set out to evaluate mechanistic heterogeneity in the acquisition of resistance to daunorubicin, which is the mainstay drug of AML induction chemotherapy regimens. To do this we generated multiple daunorubicin-resistant K562 leukemia cell lines in parallel. K562 cells are derived from the pleural effusion of a patient with chronic myeloid leukemia in terminal myeloid blast crisis (8), and, unmanipulated, they undergo apoptosis in response to daunorubicin with an IC_50_ of approximately 40 nM. We selected this line in view of its extensive use as a model system by the ENCODE Consortium.

**Figure 1:**
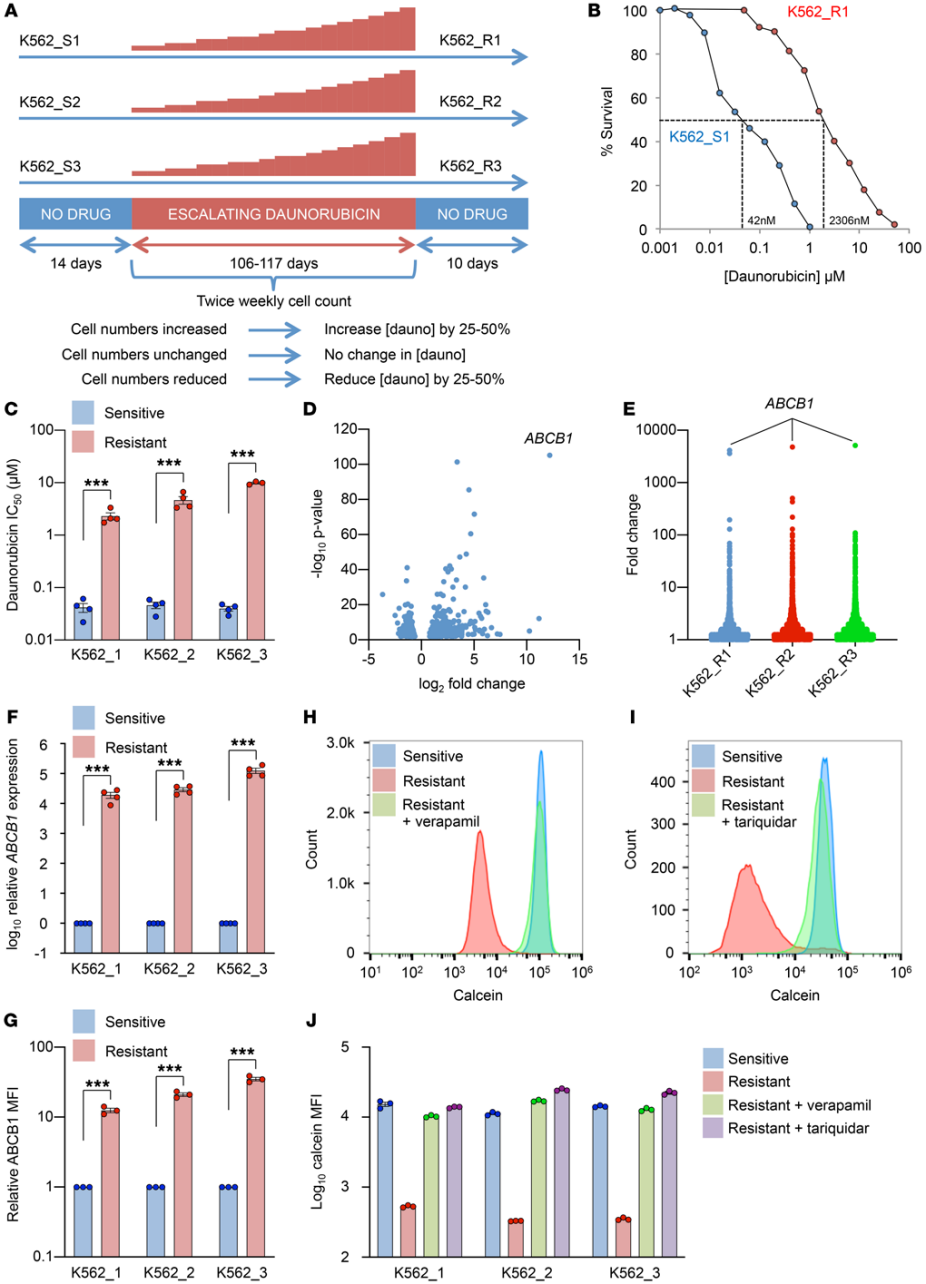
Resistance to daunorubicin due to stereotypical induction of *ABCB1*. (**A**) Outline of experiment. (**B**) Dose-response curves for sensitive and resistant lines following 72 hours of treatment with the indicated doses of daunorubicin. (**C**) Bar chart shows mean ± SEM IC_50_ values for daunorubicin for all lines (*n* = 4). ***P < 0.001 by unpaired *t* test. (**D**) Volcano plot shows differential gene expression between sensitive (K562_S1–3) and resistant (K562_R1–3) cell lines. (**E**) *ABCB1* is the most highly upregulated gene in each resistant line compared with its sensitive parental line. (**F**) Mean ± SEM fold increase in *ABCB1* expression, as determined by quantitative PCR (*n* = 4). ***P < 0.001 by unpaired *t* test. (**G**) Mean ± SEM fold increase in ABCB1 median fluorescence intensity (MFI), as determined by flow cytometry (*n* = 3). ***P < 0.001 by unpaired *t* test. (**H** and **I**) Representative flow histograms show calcein AM retention in the indicated lines in the presence or absence of verapamil 40 μM (**H**) or tariquidar 50 nM (**I**). (**J**) Summary of calcein AM retention data for all 3 line pairs for verapamil and tariquidar (*n* = 3).

Three separate vials of early-passage K562 cells were thawed and cultured separately for 2 weeks. The 3 drug-sensitive lines were designated K562_S1–3, and aliquots were cryopreserved for later use. Each line was then exposed to escalating doses of daunorubicin in continuing culture until they were able to expand in 500 nM (Figure 1A). Resistant lines were designated K562_R1–3, and the time taken to acquire this level of resistance was 106 days (K562_R1 and R3) or 117 days (K562_R2). The daunorubicin IC_50_ values were 2.3 μM, 4.7 μM, and 9.9 μM, respectively, with 55-fold, 101-fold, and 249-fold increases versus drug-sensitive lines K562_S1–3, respectively (Figure 1, B and C).

To evaluate changes in gene expression, we performed RNA sequencing. To avoid detecting transient changes in gene expression associated with recent daunorubicin exposure or contamination with apoptotic cells, each line was propagated for a further 10 days without daunorubicin prior to RNA extraction. RNA sequencing was performed using a single replicate for each sensitive line (K562_S1–3) and 2 replicates for each resistant line (K562_R1–3). When each drug-resistant line was compared with the sensitive lines, the most highly upregulated protein coding gene in each case was *ABCB1* (mean 4700-fold) even though the lines had been cultured separately from one another for at least 4 months (Figure 1, D and E, and Supplemental Table 1; supplemental material available online with this article; https://doi.org/10.1172/JCI130809DS1). Increased *ABCB1* expression was confirmed by quantitative PCR, and this correlated well with increased cell surface ABCB1 protein (Figure 1, F and G). To confirm that the upregulated protein expression was functional, we performed fluorescent dye efflux experiments. Drug-sensitive K562_S lines did not efflux calcein acetoxymethyl (calcein AM), whereas drug-resistant K562_R lines exhibited robust drug efflux (Figure 1, H and I). Efflux was completely reversed by either verapamil (a nonspecific ABC transporter substrate) or tariquidar (a highly specific inhibitor of ABCB1) (5). This confirmed that all drug efflux was due to ABCB1 (Figure 1J). No other ABC transporter gene was upregulated more than 2.5-fold in resistant cells (Supplemental Table 1). Thus even when chemoresistance is induced in separate lines, the mechanism of acquisition (i.e., *ABCB1* upregulation) is stereotypical.

### Daunorubicin-resistant leukemia cells express a common integrated stress response–like gene signature.

**Figure 2:**
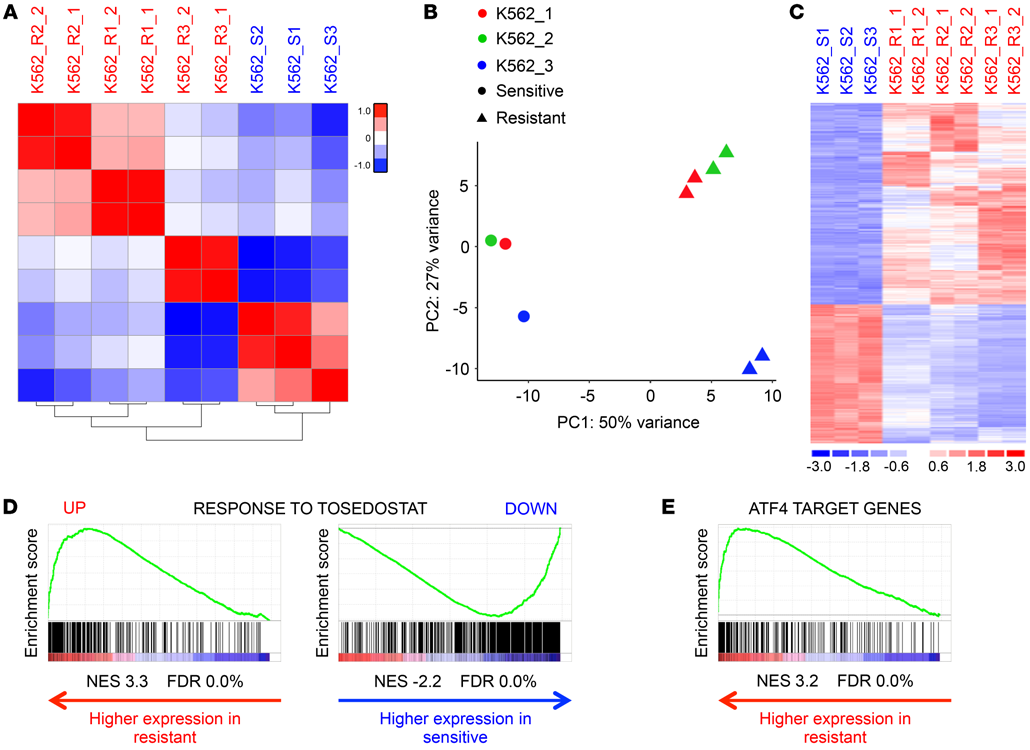
Daunorubicin-resistant leukemia cells express a common ISR-like gene signature. (**A**) Similarity matrix and hierarchical clustering of samples by differential gene expression. (**B**) Principal component (PC) analysis of gene expression from all sensitive and resistant cell lines. (**C**) Heatmap shows differentially expressed genes (223 upregulated and 154 genes downregulated; *t* test, *P* < 0.01, fold change >2 or <0.5). (**D** and **E**) Gene set enrichment analysis plots.

Unsupervised hierarchical clustering analysis, using cosine distance and average linkage, of 5953 expressed protein coding genes revealed that transcriptomes of sensitive and resistant lines differed substantially from one another (Figure 2A). Interestingly, principal component analysis revealed differences in the transcriptome of K562_S3 compared with both K562_S1 and K562_S2 (PC2), which were preserved as cells developed resistance (Figure 2B). PC1 accounted for 50% of the variance and defined the transition from sensitive to resistant in each case. Differential gene expression analysis identified 223 and 154 genes as significantly upregulated or downregulated, respectively (*t* test, *P* < 0.01, fold change >2 or <0.5) (Figure 2C and Supplemental Table 2). Among the upregulated gene set there was significant enrichment for Gene Ontology Biological Process terms, reflecting cellular stress including “response to endoplasmic reticulum stress” and “endoplasmic reticulum unfolded protein response”; among the downregulated gene set there was enrichment for “rRNA processing” and “mRNA splicing, via spliceosome” (9). Gene set enrichment analysis (GSEA) revealed that, of the 2414 curated gene sets from the Molecular Signatures Database tested (version 6.2) (10), those reflecting the response of HL-60 promyelocytic leukemia cells to the aminopeptidase inhibitor tosedostat (11), and arterial endothelial cells to hypoxia (12), were the most significantly enriched among both up- and downregulated genes in daunorubicin-resistant versus sensitive K562 cells (Figure 2D and Supplemental Figure 1A). Tosedostat is an aminopeptidase inhibitor that induces intracellular amino acid deprivation and consequent activation of the integrated stress response (ISR). Likewise, hypoxia activates the ISR by impairing disulfide bond formation, causing protein misfolding and endoplasmic reticulum stress (13).

To identify candidate regulators of high-level *ABCB1* transcription, and more generally the associated ISR-like transcriptional program, we identified transcription factor genes upregulated in resistant versus sensitive cells (Table 1 and Supplemental Tables 1 and 2). The most highly expressed was *ATF4*. Others included ATF4-bound transcriptional targets such as *ATF3*, *XBP1*, and *CEBPB* (Supplemental Table 2) or ATF4 binding partners including *JUN*, *JUNB*, *CEBPB*, *CEBPG*, *DDIT3*, and *ATF3* (14, 15). Consistent with ATF4 being a core driver of the upregulated ISR-like transcriptional program, GSEA demonstrated highly significant enrichment for ATF4 target genes among genes upregulated in daunorubicin-resistant versus sensitive K562 cells (Figure 2E). In this analysis, ATF4 target genes were those identified as genes closest to the strongest 500 ATF4 binding peaks identified by ChIP-Seq in K562 cells (Supplemental Table 2 and refs. 14, 16). Similar analyses using sets of genes located closest to the 500 strongest CEBPB, CEBPG, ATF3, JUN, or JUNB binding peaks in K562 cells also revealed significant enrichment in daunorubicin-resistant K562 cells (Supplemental Table 2 and refs. 14, 16). Notably, however, enrichment scores were lower than for the analysis using ATF4 target genes (Supplemental Figure 1B). All together these data demonstrate that the acquisition of an ABCB1-dependent daunorubicin-resistant cellular state in myeloid leukemia cells is associated with sustained upregulation of an ISR-like transcriptional program, with the transcription factor ATF4 at its core.

### Expression of ABCB1 is regulated by a stress-responsive enhancer.

Despite its clinical significance as a critical regulator of chemoresistance, knowledge of the transcriptional control of *ABCB1* is incomplete. Constitutive expression of its promoter requires motifs within 250 bp of the transcription start site that facilitate binding of nuclear factor-Y and SP1, and promoter binding sites for EGR1, WT1, HIF1A, CEBPB, FOXO factors, and TCF7 have been reported (17). TP53 may repress or activate the *ABCB1* promoter depending on whether it is wild-type or mutant; promoter DNA methylation represses *ABCB1* expression; and genetic translocations may activate *ABCB1* expression through juxtaposition of the native promoter to that of more active but unrelated genes (4). *ABCB1* expression may also be induced by stressful stimuli, and roles for the AP-1 transcription factor family and nuclear factor-κB have been suggested, but supporting data are indirect (4, 17). There is no knowledge as to whether *ABCB1* is regulated by enhancer elements, and, if so, which factors control these.

**Figure 3:**
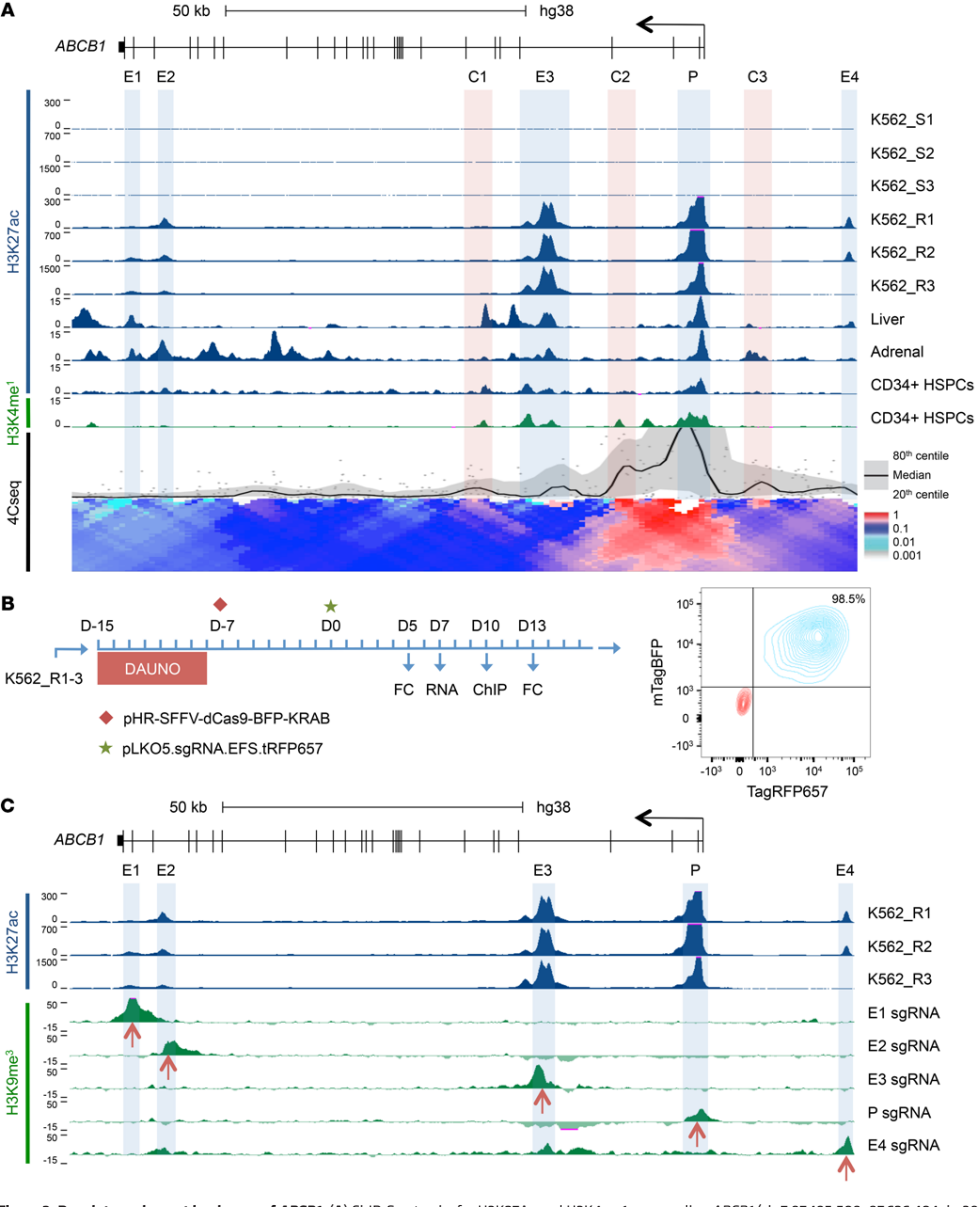
Regulatory element landscape of *ABCB1*. (**A**) ChIP-Seq tracks for H3K27Ac and H3K4me1 surrounding *ABCB1* (chr7:87,495,508–87,626,404; hg38) in the indicated human cells and tissues, including CD34^+^ hematopoietic stem and progenitor cells. Putative enhancers (E1–E4) are highlighted in blue. Lower track shows a local contact profile generated from 4C sequencing of K562_R1 using a viewpoint centered on the *ABCB1* promoter. Regions of contact that do not contain an active enhancer in K562_R1–3 are highlighted in red (C1–C3). (**B**) Experimental outline (left); and representative flow cytometry plot (right) showing double-positive population (blue; K562_R1 BFP^+^RFP^+^) and negative control population (red). FC, flow cytometry. (**C**) H3K9me3 ChIP-Seq tracks for each sgRNA. Signal from empty vector was subtracted to show only histone methylation resulting from presence of the guide. Red arrows indicate the position of the target sequence. H3K27Ac ChIP-Seq tracks from **A** are included for reference.

To identify candidate *ABCB1* enhancer elements, we performed ChIP-Seq for H3K27 acetylation, a histone modification that marks active enhancers (18), using sensitive (K562_S1–3) and resistant (K562_R1–3) lines. We searched a 2-Mb region centered on *ABCB1* for differentially acetylated regions in resistant versus sensitive lines; the great majority of *cis*-regulatory elements lie within 1 Mb of target genes (19). Consistent with the dramatic increase in transcription, there was strong promoter acetylation in drug-resistant lines, which was not observed in sensitive lines. In addition, we identified 4 acetylation peaks, designated E1–E4, in intronic sequences of *ABCB1* (E1–E3) or upstream of the promoter (E4) in resistant but not sensitive cell lines (Figure 3A).

Using H3K27Ac ChIP-Seq data from ENCODE (14), we also searched for candidate enhancer elements in normal liver and adrenal gland, the tissues with the highest constitutive levels of *ABCB1* expression (Figure 3A). The pattern was tissue-specific, although putative *ABCB1* enhancers from K562_R1–3 lines were acetylated in liver (E1 and E3) or adrenal gland (E1, E2, and E3). Normal human CD34^+^ hematopoietic stem and progenitor cells (HSPCs) express intermediate levels of *ABCB1*, and H3K27 acetylation of E3 was observed (Figure 3A). Interestingly, E3 and four additional sites were marked by H3K4 monomethylation in CD34^+^ HSPCs, a histone modification that marks poised as well as active enhancers.

To determine the nature of candidate regulatory element contacts, we next performed 4C sequencing in drug-resistant cells with a viewpoint centered on the *ABCB1* promoter. There were particularly strong interactions between E3 and E4 and the promoter, and lower-level interactions between E1 and E2 and the promoter (Figure 3A and Supplemental Figure 2A). Strong contact was also observed between 3 additional regions, termed C1, C2, and C3, and the promoter. C1 is H3K4-monomethylated and weakly H3K27-acetylated in CD34^+^ HSPCs, and strongly acetylated in liver; C2 is H3K4-monomethylated in CD34^+^ HSPCs; and C3 is acetylated in the adrenal gland. These observations suggest that C1–C3 may exhibit tissue-specific enhancer activity, although the presence of constitutive contact with the promoter in K562_R cells may be explained by C1 being bound by CTCF and cohesin (Supplemental Figure 2B). The reason for contacts between C2, C3, and the promoter was not apparent. Thus, the *ABCB1* promoter exhibits a network of physical contacts with nearby enhancers in drug-resistant K562 leukemia cells.

**Figure 4:**
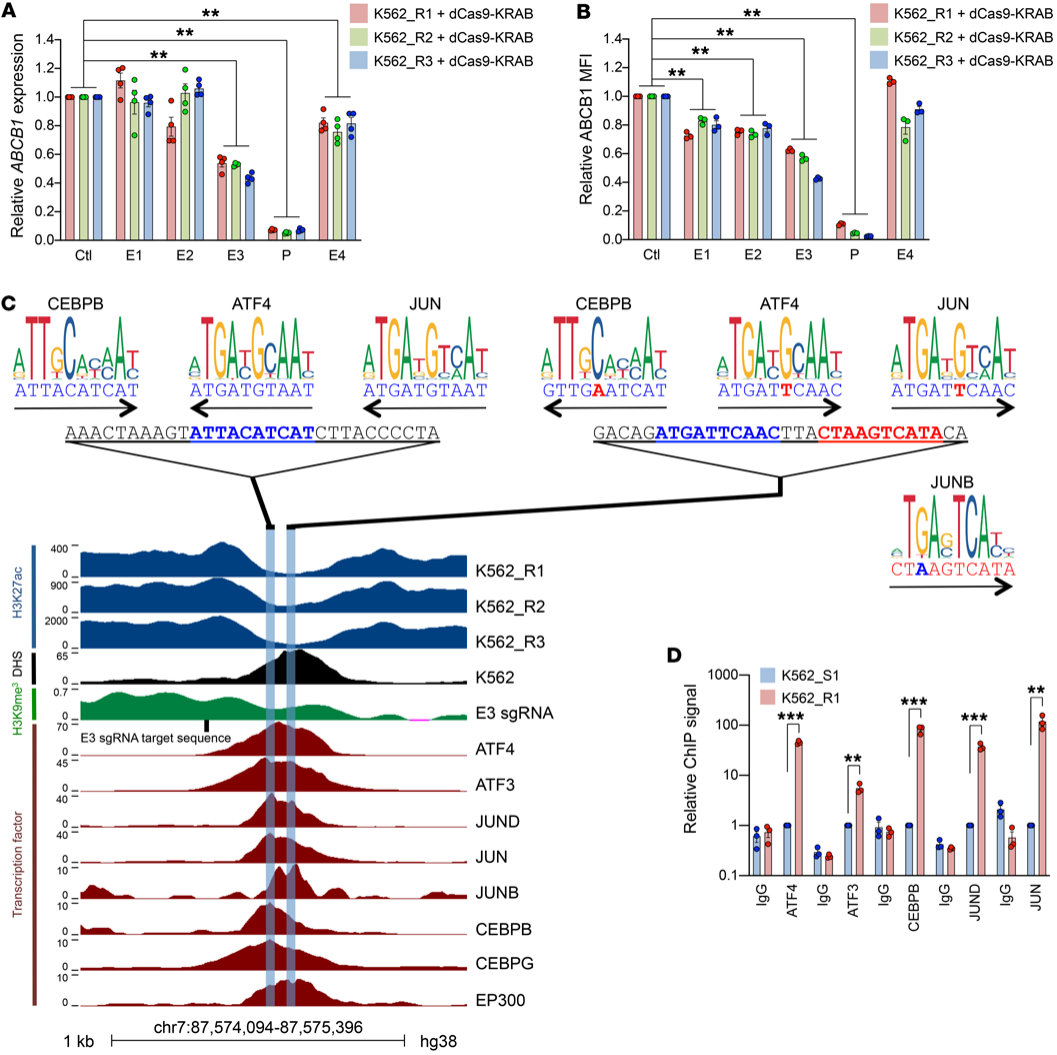
Expression of *ABCB1* is regulated by a stress-responsive enhancer. (**A**) Mean ± SEM *ABCB1* expression by quantitative PCR in dCas9-KRAB^+^ resistant cell lines (K562_R1–3) expressing sgRNAs targeting the indicated putative enhancer elements (E1–E4) or the promoter (P), relative to control cells expressing a nontargeting guide (Ctl). **P < 0.01 by 1-way ANOVA with Tukey’s post hoc test (*n* = 4). (**B**) As for **A**, but with mean ± SEM ABCB1 median fluorescence intensity (MFI) by flow cytometry. **P < 0.01 by 1-way ANOVA with Tukey’s post hoc test (*n* = 3). (**C**) ChIP-Seq tracks for H3K27Ac, H3K9me3 (our data), and the indicated transcription factors in K562 cells (ENCODE); and DNase-Seq (ENCODE) at the E3 enhancer. Sites of AP-1 binding motifs are indicated. (**D**) Mean ± SEM relative ChIP PCR signal for the indicated transcription factors for K562_R1 and K562_S1 using primers for the E3 enhancer. **P < 0.01, ***P < 0.001 by unpaired *t* test (*n* = 3).

To confirm that putative enhancers were functional, we next performed targeted silencing using a CRISPR-dCas9-KRAB system. We designed multiple sgRNAs for each region (Supplemental Figure 3, A–D) and screened them in K562_R3 cells, using loss of cell surface ABCB1 expression or increased calcein AM retention as a measure of activity (Supplemental Figure 3, E and F). The most active guides were then selected for use in all resistant cell lines. K562_R1–3 cells were dual-infected with pHR-SFFV-dCas9-BFP-KRAB and pLKO5.sgRNA.EFS.tRFP657, the latter expressing an sgRNA targeting an enhancer or the promoter, or a nontargeting control (Figure 3B). We used ChIP-Seq for H3K9 trimethylation to confirm that silencing was discrete and accurate: induced regions of heterochromatin ranged in size from 3 to 8 kb, were centered on the target sequence for each guide, and did not target the promoter, even where 4C-Seq had shown the enhancer region to be in close physical proximity (Figure 3C). Quantitative PCR and flow cytometry assessment of the effect of *ABCB1* promoter silencing revealed substantial repression of transcription (Figure 4, A and B). The enhancer silencing experiments revealed either modest or no significant contribution to ABCB1 expression from E1, E2, and E4. The most extensive reductions in expression of *ABCB1* transcripts and protein were observed following silencing of E3, demonstrating that this was the most active enhancer, consistent with its high level of H3K27 acetylation and close contact with the promoter.

Within E3 is a DNase I–hypersensitive site (Figure 4C and ref. 14). Motif analysis of the 30-bp sequence revealed consensus binding sites for several of the transcription factors upregulated in drug-resistant cells, including ATF4, JUN, and CEBPB (Tables 1 and 2 and Figure 4C). We used ENCODE ChIP-Seq data from unmanipulated K562 cells to characterize binding of those factors to each enhancer (Figure 4C and Supplemental Figure 4). Data sets were available for 6 of the 12 factors upregulated in resistant cells (Supplemental Table 3), all of which were bound to the E3 enhancer, suggesting it to be stress-responsive (Figure 4C). There was some modest ATF3 and ATF4 binding at E1 and adjacent to E4. Critically, binding of AP-1 transcription factors to the promoter was absent (Supplemental Figure 4). Interestingly, E2 exhibited binding of TAL1 and GATA2, key hematopoietic stem cell (HSC) transcription factors that are active in AML and associated with poor clinical outcome (20). To confirm the ENCODE data and to determine whether there was increased binding of stress-responsive transcription factors at E3 in drug-resistant cells, we performed ChIP PCR. We observed significant increases in the binding of ATF4, ATF3, CEBPB, JUND, and JUN to E3 in K562_R1 compared with K562_S1 cells (Figure 4D). These data together demonstrate that acquisition of daunorubicin resistance is associated with activation of a stress-responsive, AP-1–bound enhancer element in intron 4 of *ABCB1*.

### Dynamic induction of ABCB1 by diverse cellular stressors.

**Figure 5:**
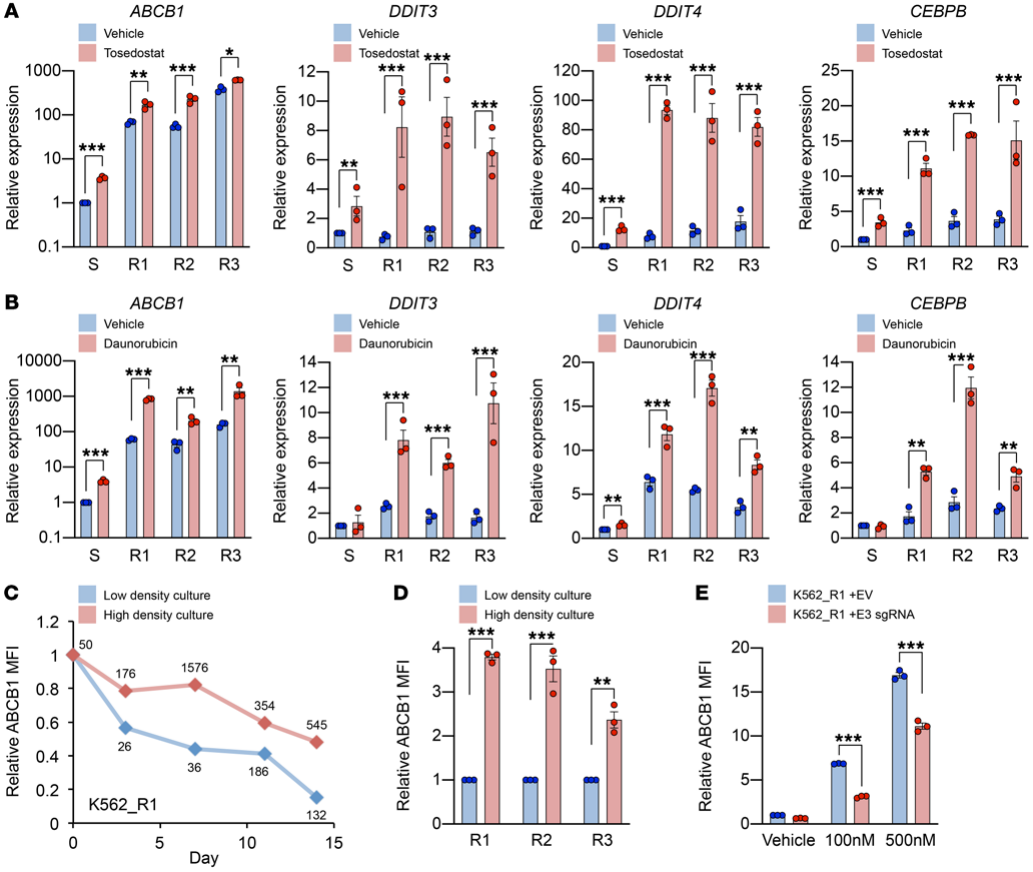
Dynamic induction of *ABCB1* by diverse cellular stressors. (**A** and **B**) Mean ± SEM expression of the indicated genes by quantitative PCR relative to a fresh aliquot of unmanipulated drug-sensitive K562 cells (*n* = 3) following exposure to tosedostat (50 μM) for 48 hours (**A**) or daunorubicin (100 nM for sensitive or 500 nM for resistant lines) for 72 hours (**B**). *P < 0.05, **P < 0.01, ***P < 0.001 by unpaired *t* test. (**C**) ABCB1 mean fluorescence intensity (MFI) over time in K562_R1 cells maintained in high- or low-density culture. Numbers indicate cell density (K/mL). (**D**) ABCB1 MFI in K562_R1–3 following 14 days of high- or low-density culture (*n* = 3). **P < 0.01, ***P < 0.001 by unpaired *t* test. (**E**) Mean ± SEM ABCB1 MFI in dCas9-KRAB^+^ resistant cells (K562_R1) expressing either an E3-targeting sgRNA or a nontargeting sgRNA (EV) following 7 days of exposure to the indicated dose of daunorubicin (*n* = 3). ***P < 0.001 by unpaired *t* test.

To explore further the relationship between cell stress and expression of *ABCB1*, but over a shorter time scale, we induced intracellular amino acid depletion using the aminopeptidase inhibitor tosedostat (11). Tosedostat is able to induce cellular stress in both sensitive and resistant cells because it is not an ABCB1 substrate subject to cellular extrusion in ABCB1^hi^ cells (Supplemental Figure 5A). There was significant upregulation of *ABCB1* expression in all K562 lines after 48 hours, although the absolute level of increase was far greater in drug-resistant lines (Figure 5A). Activation of the ISR upregulates ATF4 through a translational mechanism (15), so it was unsurprising that changes in *ATF4* transcript levels were modest (Supplemental Figure 5B). Instead, as a surrogate measure of ATF4 activity, we quantified expression of 3 genes that are known direct targets of ATF4: *DDIT3*, *DDIT4*, and *CEBPB* (21). Expression of all three was robustly induced by tosedostat, again with the absolute level of increase being greater in drug-resistant lines (Figure 5A). Tosedostat also induced expression of the AP-1 transcription factor *JUN* in all lines (Supplemental Figure 5C). Similar observations were made following treatment of unmanipulated early-passage K562 cells with alternate stressors: thapsigargin, which activates the ISR through blockade of the endoplasmic reticulum Ca^2+^ ATPase (Supplemental Figure 5D and ref. 22), and high-density culture (cell density of >10^6^/mL for 48 hours; Supplemental Figure 5E). Thus, diverse cellular stressors induce dynamic upregulation of *ABCB1* and other direct targets of ATF4.

Exposure of sensitive and resistant K562 lines to 100 nM and 500 nM daunorubicin, respectively, for 72 hours also induced *ABCB1* (Figure 5B). As for tosedostat, the greatest absolute levels of increase were observed in drug-resistant lines, and they correlated with significant increases in *DDIT3*, *DDIT4*, and *CEBPB* (Figure 5B). By contrast with tosedostat, the fold-change increases in *DDIT3*, *DDIT4*, and *CEBPB* induced by 500 nM daunorubicin were lower, and increased expression of *ATF4* was not observed, suggesting that daunorubicin may be a somewhat less efficient activator of the ISR pathway (Supplemental Figure 5F). Daunorubicin also induced expression of *JUN* in resistant lines (Supplemental Figure 5G). The differences in response to both tosedostat and daunorubicin of drug-sensitive versus drug-resistant K562 lines are in keeping with the observed enhancer remodeling at the *ABCB1* locus induced by prolonged (>100 days) daunorubicin exposure.

These data demonstrate that brief daunorubicin exposure also induces ATF4 target gene expression, including *ABCB1*. Importantly, ABCB1 expression in daunorubicin-resistant K562 lines was dynamic and diminished over time if cells were not continuously exposed to drug (Figure 5C). Loss of ABCB1 expression was more pronounced when cells were propagated at low density (<200,000/mL), emphasizing the need for rigorous control of cell culture conditions when performing stress experiments. Even modest elevations of cell density (>200,000/mL) were sufficient to cause significant increases in ABCB1 in comparison with low-density controls (Figure 5, C and D). Re-exposure of K562_R1–3 cells to daunorubicin (100 or 500 nM for 7 days) following a 24-day daunorubicin-free period of culture led to a dose-dependent reestablishment of *ABCB1* expression, an effect that was dependent on the activity of the ATF4-bound E3 enhancer, since it was attenuated when E3 was silenced with dCas9-KRAB (Figure 5E). All together these data demonstrate that expression of the daunorubicin drug export pump ABCB1 is dynamically regulated in leukemia cells though the ATF4-bound E3 enhancer.

### Daunorubicin activates a stress-responsive ABCB1 enhancer in primary AML cells.

**Figure 6:**
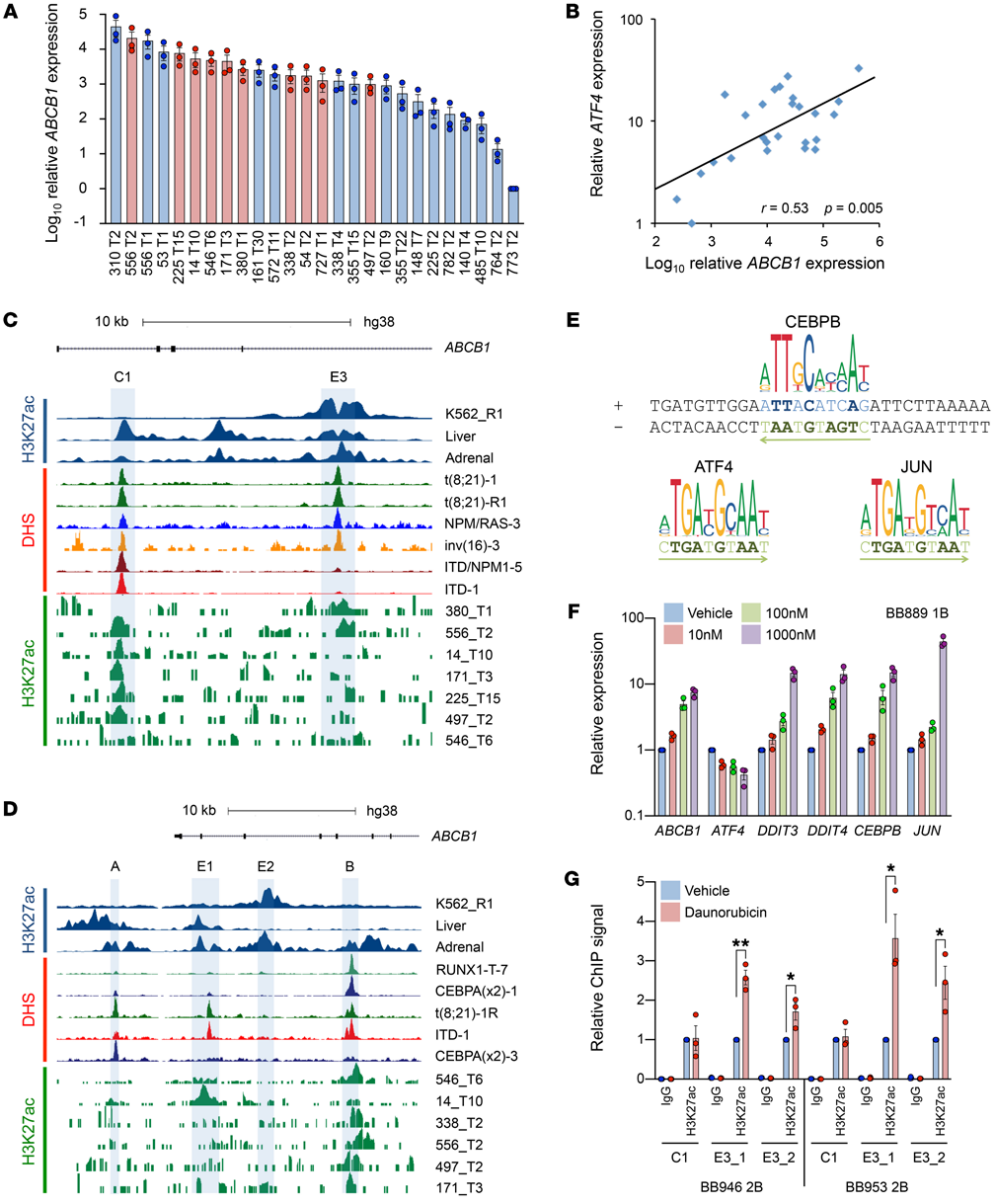
Daunorubicin activates a stress-responsive *ABCB1* enhancer in primary AML cells. (**A**) *ABCB1* expression by quantitative PCR in primary AML samples (*n* = 3). H3K27Ac ChIP-Seq was performed on the samples highlighted in red. (**B**) Correlation of *ATF4* and *ABCB1* expression; *r* = Pearson product-moment correlation coefficient. (**C** and **D**) ChIP-Seq (our data) and DNase-Seq tracks (23) surrounding C1 and E3 (chr7:87,561,371–87,579,610; hg38) (**C**) and A, B, E1, and E2 (chr7:87,494,187–87,522,854; hg38) (**D**) from the indicated cell lines, human tissues (ENCODE), and primary AML samples. (**E**) Transcription factor binding motifs identified at the center of site B. (**F**) Mean ± SEM relative expression of the indicated genes following exposure of fresh primary AML blast cells to the indicated doses of daunorubicin for 18 hours (*n* = 3). BB numbers indicate Biobank identifier. (**G**) Mean ± SEM relative ChIP PCR signal for H3K27Ac using fresh primary AML blast cells exposed to 1000 nM daunorubicin or vehicle for 18 hours (*n* = 3). Data from 2 patients (BB946 and BB953) are shown. PCR was performed using 2 primer sets for the E3 enhancer (E3_1 and E3_2) and 1 for the CTCF binding site C1. *P < 0.05, **P < 0.01 by unpaired *t* test. BB numbers indicate Biobank identifier.

We next examined *ABCB1* enhancer accessibility and usage in primary AML. We identified cases of relapsed or refractory AML from Manchester Cancer Research Centre’s Tissue Biobank with high *ABCB1* expression by quantitative PCR (Figure 6A and Supplemental Table 4) and performed ChIP sequencing for H3K27Ac in high-expressing cases where sufficient cryopreserved bulk blast cells were available (red bars in Figure 6A). We also made use of a recently published DNase-Seq primary AML data set (23). Quantitative PCR analysis revealed that *ATF4* expression correlated significantly with *ABCB1* (Figure 6B; *r* = 0.53, *P* = 0.005). Considering the genomic region encompassing the coding sequence of *ABCB1* and sequences 20 kb upstream and 10 kb downstream, we identified 5 DNase I–hypersensitive sites (DHSs) (in addition to the DHS observed at the promoter) in multiple cases of AML (Figure 6, C and D). These included E1 and E3 (accessible in 12 of 36 and 13 of 36 primary AML cases, respectively), which became strongly acetylated in drug-resistant K562 cells, and the CTCF binding site C1 (accessible in 32 of 36 primary AML cases) (Figure 3A and Figure 6, C and D). Regions E2 and E4 (Figure 6D and data not shown) were not accessible. Two additional sites (A and B; accessible in 13 of 36 and 14 of 36 cases, respectively), which were not acetylated in drug-resistant K562 cells, were also DNase I–hypersensitive. DHS site B was adjacent to other confirmed *ABCB1* enhancers (E1 and E2; Figure 6D) and was acetylated in ABCB1-expressing adrenal tissue. Importantly, this site also contains binding motifs for ATF4, JUN, and CEBPB, suggesting that it too may serve as a stress-responsive enhancer (Figure 6E). Across the totality of primary AML samples profiled by Assi et al. (23), 6 of 36 exhibited DHS at both B and E3 sites, 8 of 36 at B only, 7 of 36 at E3 only, and 15 of 36 at neither B nor E3. Together these data show that stress-responsive regulatory elements are accessible in bulk primary AML cells. Our own H3K27Ac ChIP-Seq analyses further demonstrated that *ABCB1*-expressing samples exhibited peaks of acetylation surrounding these sites: of the 10 samples analyzed, 4 had discernible H3K27Ac peaks at B only, 2 at E3 only, 1 at both, and 3 at neither. In 1 case there was a peak of acetylation at A. Thus, in a substantial proportion of primary AML cases, stress-responsive *ABCB1* regulatory elements are accessible and active.

To determine whether primary AML cells respond to stress in a similar manner to drug-resistant K562 cells, we exposed fresh bulk primary AML blasts from bone marrow or blood (Supplemental Table 4) to daunorubicin (10 nM, 100 nM, and 1000 nM) for 18 hours. We observed dose-dependent induction of ATF4 target genes *ABCB1*, *DDIT3*, *DDIT4*, *CEBPB*, and *JUN*, although, as before, changes in *ATF4* transcripts were modest or absent (Figure 6F and Supplemental Figure 6A). It was of note that this response was not observed when similar analyses were performed using cryopreserved AML samples following a freeze-thaw cycle (Supplemental Figure 6B). Vehicle-treated freeze-thawed samples exhibited substantially higher levels of *ATF4* and *DDIT3* compared with vehicle-treated fresh samples (Supplemental Figure 7A), suggesting that the freeze-thaw process activates cellular stress pathways consequently obscuring the response to daunorubicin exposure. Two additional fresh primary AML samples were treated with 1000 nM daunorubicin or vehicle for 18 hours (Supplemental Figure 7B) and subjected to ChIP PCR for H3K27Ac surrounding E3 (Figure 6G). Significant increases in acetylation were observed, confirming acute stress–induced regulation of E3. By contrast, daunorubicin had no effect on the acetylation of the CTCF binding site C1 (Figure 6G).

We also assessed the effect of daunorubicin exposure on 2 other ABC transporter genes previously associated with chemoresistance in AML (3). *ABCG2* expression increased significantly in 4 of 5 fresh samples following daunorubicin exposure, but absolute levels of expression were very low as judged by cycle threshold (Supplemental Figure 7C). Induction was not observed in 3 of 4 freeze-thawed samples (Supplemental Figure 7D). *ABCC1* was more highly expressed and its expression increased significantly in all fresh samples (Supplemental Figure 7E), with responses again smaller or absent in freeze-thawed samples (Supplemental Figure 7F). The change in expression of these efflux pumps in response to daunorubicin mirrors that of *ABCB1*, suggesting regulation by similar mechanisms. Interestingly, ENCODE data in unmodified K562 cells shows intronic binding of CEBPB, CEBPG, JUND, JUN, ATF4, and ATF3 within *ABCC1*, suggesting that this efflux pump may also be stress-responsive (Supplemental Figure 8 and ref. 14).

*ABCB1* is also expressed in normal HSCs and downregulated during differentiation. Indeed, extrusion of rhodamine 123 or Hoechst 33342 by ABCB1 has been used to identify long-term repopulating HSCs (24). HSCs also make use of the ISR and ATF4 to protect against homeostatic cellular stress and to preserve the integrity of the stem cell pool (25). Given this account of an adaptive, prosurvival ISR signature in HSCs, we examined the expression of K562 resistance–associated transcription factor genes across normal hematopoiesis (26). As previously described, *ABCB1* expression diminished as cells differentiated, with the highest expression seen in early HSCs (Supplemental Figure 9A). *ATF4* expression followed a similar pattern and was highly correlated with *ABCB1* (Supplemental Figure 9, A–C; *r* = 0.91, *P* < 0.001). Given the predominantly translational regulation of ATF4, we also studied its transcriptional target *DDIT3*; changes in expression correlated even more closely with that of *ABCB1* (Supplemental Figure 9, A–C; *r* = 0.95, *P* < 0.001). Indeed, all of the transcription factorsthat were upregulated in daunorubicin-resistant K562 cells were significantly correlated with *ABCB1* expression across normal hematopoiesis (Supplemental Figure 9B). Reflecting this observation, GSEA revealed highly significant enrichment of expression of the 223 genes upregulated in drug-resistant versus sensitive K562 cells in normal hematopoietic stem cell/multipotent progenitor versus downstream myeloid progenitor populations (Supplemental Table 2 and Supplemental Figure 9D), suggesting a common gene expression program driven by adaptive prosurvival stress signaling. Analysis of H3K27Ac and H3K4me1 ChIP-Seq and DNase-Seq data (ENCODE) from normal CD34^+^ HSPCs confirmed that regulatory elements A, E1, E3, and, to a lesser extent, B were accessible in CD34^+^ HSPCs and marked by H3K4 monomethylation and, in the case of E3, by H3K27 acetylation (Supplemental Figure 9E).

These data demonstrate the close link between expression of a stress-responsive genetic program and resistance to daunorubicin through upregulation of *ABCB1*; they further demonstrate that chemotherapy treatment with daunorubicin activates a stress-responsive enhancer and induces upregulation of a drug resistance mechanism in AML blast cells that may contribute to therapeutic failure and disease relapse.

### Activation of an ISR-like response facilitates escape from ABCB1 inhibition.

Pharmacologic inhibitors of ABCB1 have been tested in clinical trials as adjuncts to AML therapy, but without significant success (3). Trials of the third-generation inhibitor tariquidar used doses of 2 mg/kg (resulting in plasma concentrations of ~4 nM), based on maximal inhibition of rhodamine 123 efflux in CD56^+^ NK cells, which exhibit relatively high, stable levels of ABCB1 expression (5). Our observation of dynamic, stress-responsive *ABCB1* expression raised a question of whether the dose of ABCB1 inhibitors used to inhibit steady-state cells might be ineffective under conditions of cellular stress.

**Figure 7:**
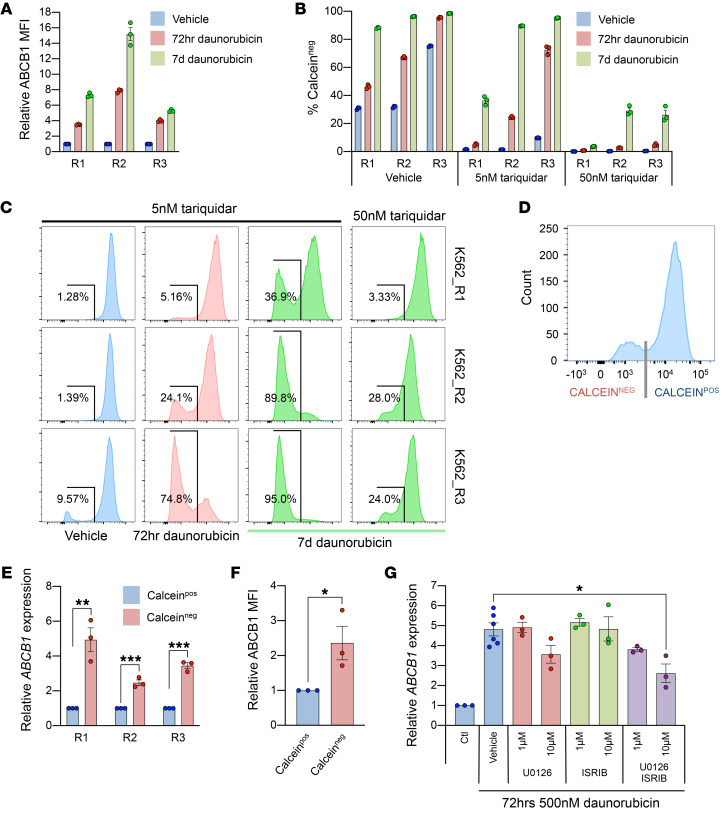
Activation of an ISR-like response facilitates escape from ABCB1 inhibition. (**A**) Mean ± SEM relative ABCB1 median fluorescence intensity (MFI) in K562_R1–3 following exposure to 500 nM daunorubicin or vehicle for 72 hours or 7 days (*n* = 3). (**B**) Proportion of cells that are calcein AM^–^ following exposure of K562_R1–3 to the indicated conditions as determined by flow cytometry (*n* = 3). (**C**) As for **B**, but showing individual flow histograms for each of the indicated conditions. (**D**) Experimental outline depicting FACS of calcein AM^–^ and calcein AM^+^ populations. (**E**) Mean ± SEM *ABCB1* expression by quantitative PCR of calcein AM^–^ and calcein AM^+^ populations (*n* = 3). **P < 0.01, ***P < 0.001 by unpaired *t* test. (**F**) As for **E**, but with relative ABCB1 MFI by flow cytometry (*n* = 3). (**G**) Mean ± SEM *ABCB1* expression by quantitative PCR in K562_R1 following exposure to 500 nM daunorubicin or vehicle for 72 hours with the indicated inhibitors (*n* = 3–6). *P < 0.05 by 1-way ANOVA with Tukey’s post hoc test (*n* = 3).

We re-exposed drug-resistant K562 cells (lines R1–3) that had been cultured without daunorubicin for 24 days to 500 nM daunorubicin or vehicle for 72 hours and assessed the ability of 5 nM tariquidar to inhibit efflux of calcein AM. As expected, re-exposure of cells to daunorubicin further induced ABCB1 expression (Figure 7A). Concomitant treatment of cells with 5 nM tariquidar abolished calcein AM efflux in vehicle-treated cells, but in daunorubicin–re-exposed cells, where ABCB1 had been further induced, in each case a population of cells was observed that failed to retain calcein AM. This demonstrates continued activity of ABCB1 drug efflux in a subpopulation of cells despite exposure of the cell population to levels of tariquidar approximating those achieved in clinical trials (Figure 7, B and C). This effect became yet more apparent when daunorubicin exposure was extended to 7 days but could be overcome by increasing of the concentration of tariquidar (Figure 7, A–C), indicating that the effect was due to differential expression of ABCB1. We confirmed this by flow-sorting tariquidar-treated K562_R1–3 cells into calcein AM^–^ and calcein AM^+^ populations and evaluating ABCB1 expression (Figure 7, D–F). Similar observations were made when K562_R1–3 cells were exposed to tosedostat, demonstrating that this effect was not specific to daunorubicin and was likely consequent upon activation of an ISR-like program (Supplemental Figure 10, A–C). To identify an approach to overcome the phenomenon of daunorubicin- and stress-induced escape from ABCB1 inhibition, we evaluated stress pathway inhibitors. U0126 antagonizes AP-1 target gene transcription via inhibition of MEK1/2, and ISRIB (integrated stress response inhibitor) antagonizes the consequences of eIF2a phosphorylation through a mechanism involving binding of eIF2B to restore normal translation of factors including ATF4 (27, 28). Treatment with 10 μM of U0126 was suggestive of reduced *ABCB1* induction in K562_R1 exposed to 500 nM daunorubicin for 72 hours compared with vehicle (Figure 7G). While ISRIB alone did not have an effect, combined treatment with U0126 led to significant dose-dependent suppression of *ABCB1* induction (Figure 7G).

Thus daunorubicin- and stress-induced acute induction of *ABCB1* can overcome pharmacologic inhibition of ABCB1, leading to leukemia cell survival, and this can, at least in part, be mitigated by concomitant treatment of cells with inhibitors of stress signaling.

## Discussion

Efflux of chemotherapeutic agents by ABCB1 is an important cause of treatment failure in human cancer. High expression levels may be an intrinsic feature of the cell type, or due to promoter translocations between *ABCB1* and genes with strong constitutive expression, such as those found in patients with breast or ovarian cancer who relapse after prior therapy (29, 30). We found that primary AML cells displayed dynamic expression of *ABCB1*, suggesting physiological regulation rather than control by constitutively active captured promoters. Our analyses reveal the network of enhancers that controls intrinsic expression of *ABCB1* in human leukemia cells; the gene is a direct target of the transcription factor ATF4, which is activated through a chemotherapy-induced cellular stress response.

Drug resistance in cancer can arise through multiple mechanisms, including genetic events and stochastic transcriptional changes in rare cells (31). We observed significant transcriptional differences in one of our cell lines (K562_3) that persisted as the line acquired resistance. These differences are likely due to the genetic and transcriptional divergence that frequently accompanies the propagation of cancer cell lines (32). Indeed, this was our primary motivation for creating 3 independent resistant cell lines, and it is significant that in spite of these differences all lines developed daunorubicin resistance through induction of *ABCB1*.

For cancer cells to survive they must adapt to stressful stimuli. The ISR is activated by endoplasmic reticulum stress, hypoxia, amino acid deprivation, and oxidative stress, common consequences of uncontrolled proliferation and outgrowth of the vascular supply. The transcription factor ATF4 is a critical effector of the ISR and is highly expressed in many cancers as a result of extrinsic stress or direct activation by constitutive oncogene expression (33). During stress it is efficiently translated as a result of eIF2α phosphorylation, permitting heterodimer formation with transcription factors such as JUN, FOS, and CEBPB, and binding of transcriptional targets (15, 34). We found that adaptation of leukemic cells to prolonged daunorubicin exposure (>100 days) involved expression of an ATF4-centered, ISR-like transcriptional program that led to sustained upregulation of *ABCB1*. ATF4 and its interaction partners bind a stress-responsive enhancer in intron 4, suggesting that this element responds specifically to stress signaling with an adaptive, prosurvival output. The dramatic differences in histone acetylation surrounding, in particular, enhancer E3 are indicative of enhancer remodeling, although the molecular mechanisms underlying this process remain unclear. It is also unclear how the various active enhancers cooperate to regulate transcriptional activity at the promoter. Uncovering these mechanisms would be of great interest, not least because the adaptive molecular changes surrounding E3 appear to serve as the basis for the epigenetic memory of prior cellular stress, at least as far as expression of *ABCB1* is concerned.

Previous reports of *ABCB1* responses to cellular stress have been contradictory, demonstrating induction or repression, even after exposure to the same stressor (35). These conflicting results might be consistent with the pleiotropic function of ATF4, which is able to orchestrate adaptation and survival or apoptosis depending on cellular context and the severity of the insult. Indeed, downregulation of *ABCB1* appears to precede cell death, suggesting that the gene is negatively regulated by ISR signaling where apoptosis is the result (36).

Even in the era of targeted therapies, tumor bulk continues to predict treatment failure for many cancers (37), and the total white cell count in blood at presentation is strongly predictive of outcome in AML (38). Our observation that prolonged daunorubicin exposure elicited a transcriptional response that was shared by cells exposed to amino acid deprivation or hypoxia suggests that extrinsic stress applied experimentally has similar consequences to environmental stresses experienced by cancer cells in vivo. We speculate that protracted cellular stress primes stress-responsive *ABCB1* enhancers for both strong constitutive activity and augmented responses following exposure to additional stressors, such as chemotherapy, leading to increased drug efflux: chemotherapy may induce its own chemoresistance mechanism. While steady-state expression of ABC transporters is seen only in a subset of resistant AML cases, we found that dynamic upregulation of *ABCB1* following daunorubicin exposure occurred in all fresh primary samples tested (39). Rapid adaptation to therapy may therefore represent a more common mechanism of resistance than previously appreciated, especially considering that the biopsies that provide primary material for research are seldom taken during treatment.

The importance of ABCB1 in hematopoiesis is well established. Its expression is a hallmark of hematopoietic stem cells (HSCs) and accounts for their reduced staining with Hoechst 33342. HSCs display high levels of prosurvival ISR activity, which we found to correlate with the expression of *ABCB1* and the transcription factor combination expressed in our resistant cells (25). Leukemic stem cells (LSCs) can also be identified by their capacity for ABCB1-mediated dye efflux (40, 41); LSCs occupy hypoxic bone marrow niches that may contribute to *ABCB1* expression and chemoresistance (42). Given the abundance of evidence supporting a role for ABCB1 in drug resistance, the lack of success of clinical trials of ABCB1 inhibitors is puzzling. A potential explanation is suggested by our observation that exposure of leukemia cells with primed *ABCB1* enhancers to daunorubicin leads to rapid and substantial upregulation of ABCB1, with escape of a leukemia cell subpopulation from the effects of drug efflux pump inhibition.

The emerging role of the ISR as driver of adaptation and survival in cancer has led to interest in pharmacologic manipulation of this pathway. We found that stress-induced upregulation of *ABCB1* could be mitigated by use of the MEK inhibitor U0126 alone or in combination with ISRIB, suggesting a possible therapeutic strategy for testing in early-phase trials. Given that the output of the ISR is dependent on the precise state of each cell, there is a risk that a therapy designed to promote apoptosis may inadvertently drive adaptation and survival in a subset of cells. The precise function of ABCB1 as an effector of adaptive stress signaling also needs to be defined. We also found that *ABCC1* expression was induced by daunorubicin exposure in fresh primary AML cells and that intronic regions likely bind the same transcription factors that drive *ABCB1* expression. The evolution of the ABC superfamily has involved gene duplication, and members presumably share previously unrecognized regulatory features (43). ABC transporters are also highly evolutionarily conserved, contributing to both nutrient import and multidrug resistance in bacteria (44). These pumps efflux a wide range of endogenous compounds and have been shown to influence paracrine signaling, membrane lipid composition, and cellular redox state (45). It is therefore likely that expression of *ABCB1* has physiological effects that mitigate certain forms of stress. In fact, the removal of chemotherapy from leukemia cells may simply be an unfortunate by-product of its primary function.

In summary, we show that cellular stress can drive chemoresistance through *ABCB1* enhancers, providing an explanation for the failure of clinical trials of ABCB1 inhibitors and suggesting an approach to overcome drug resistance. This study has implications for the study of resistance mechanisms more generally, as these data demonstrate that the behavior of cancer cells is highly dependent on cell context and environmental factors. Studies of cells in steady state alone may be potentially misleading.

## Methods

### Cell culture.

K562 cells were from DSMZ and were cultured in RPMI 1640 medium supplemented with 2 mM l-glutamine (Life Technologies) and 10% FBS (Sigma-Aldrich). While under drug selection, cells were counted and replated every third day. Cell lines were confirmed mycoplasma-free and authenticated by short tandem repeat DNA profiling.

### Primary AML samples.

Primary human AML samples were from the Manchester Cancer Research Centre Tissue Biobank (approved by the South Manchester Research Ethics Committee). Their use was authorized by the Tissue Biobank’s scientific subcommittee, with the informed consent of donors. For ChIP, selected samples were thawed or collected fresh and immediately cross-linked. For treatment with daunorubicin, fresh leukemic blast cells were obtained by density gradient centrifugation of bone marrow or peripheral blood. Cells were treated in α-MEM medium supplemented with 12.5% heat-inactivated FBS, 12.5% heat-inactivated horse serum, 2 mM l-glutamine, 57.2 μM β-mercaptoethanol, 1 μM hydrocortisone (Sigma-Aldrich), and IL-3, G-CSF, and TPO (all at 20 ng/mL; PeproTech).

### Reagents.

Daunorubicin, verapamil, and ISRIB were from Sigma-Aldrich; tosedostat and tariquidar were from Generon; and thapsigargin and U0126 were from Merck. Compounds were resuspended in DMSO (tosedostat, tariquidar, thapsigargin, ISRIB, U0126) or ddH20 (verapamil and daunorubicin), aliquoted, and stored at –20°C. Final DMSO concentration was less than 0.5% in all experiments.

### Cell viability assays.

5 × 10^3^ cells were plated in each well of a 96-well plate with media containing a serial dilution of daunorubicin. Plates were incubated for 72 hours at 37°C. Twenty microliters of 140 μg/mL resazurin (Sigma-Aldrich) was added to each well. Plates were then incubated for a further 4 hours and read using a POLARstar Omega plate reader (BMG Labtech).

### RNA sequencing and data analysis.

Total RNA was extracted from 5 × 10^5^ cells using QIAshredder spin columns and an RNeasy Plus Micro kit (Qiagen). Before sequencing, RNA integrity was checked using a 2100 Bioanalyzer (Agilent Technologies). Poly(A) libraries were prepared using a SureSelect Poly(A) kit (Agilent Technologies); samples were then barcoded and pooled. Sequencing was performed using a NextSeq desktop sequencing system (Illumina). A single run (400 million reads) of 75 bp paired-end sequencing produced a mean of 45.7 million reads per sample. Reads were aligned to the human genome (hg38) using STAR version 2.4.2a (46). DEseq2 was used to perform differential gene expression analysis and calculate FPKM (fragments per kilobase of transcript per million mapped reads) values for each transcript (47). Hierarchical clustering, similarity matrix, and heatmap visualizations were created using clustergrammer (48). Principal component analysis was performed using ggplot2 (49). Gene set enrichment analyses (10) were performed with GSEA version 2.0 software (http://www.broad.mit.edu/gsea) using signal-to-noise for gene ranking and 1000 data permutations. To identify ATF4, CEBPB, CEBPG, ATF3, JUN, or JUNB target genes, K562 ChIP-Seq data sets were downloaded from the ENCODE Consortium (14). The strongest peaks by pileup value were identified by Model-based Analysis of ChIP-Seq version 2 (MACS2) using default parameters (Supplemental Table 2 and ref. 50). Gene expression and *ABCB1* correlations in sorted cord blood populations were analyzed using data from Laurenti et al. (26). Raw data files for RNA sequencing are available at the Gene Expression Omnibus with the accession number GSE131825.

### Quantitative PCR.

cDNA was generated using a High Capacity Reverse Transcription kit (Applied Biosystems). Quantitative PCR reactions were performed in MicroAmp optical 384-well reaction plates and analyzed using a QuantStudio 5 PCR system (Applied Biosystems). Reactions were performed in triplicate or quadruplicate and included primers for β-actin (ACTB) as a housekeeping gene. Primers were designed using the Universal Probe Library (UPL) Assay Design Center (Roche) and purchased from Integrated DNA Technologies. Raw fluorescence data were converted to Ct values using the Thermo Fisher Cloud facility and normalized to ACTB. For primer sequences and associated probes see Supplemental Table 5.

### FACS, flow cytometry, and assessment of calcein-AM retention.

Flow cytometry was performed using an LSR II flow cytometer (BD Biosciences). A FACSAria II (BD Biosciences) was used for cell sorting experiments. FlowJo version 10.1 (BD Biosciences) was used to analyze data. To assess calcein AM retention, 5 × 10^5^ cells were resuspended in PBS containing 10 nM freshly prepared calcein AM (BioLegend) with 40 μM verapamil, 5 or 50 nM tariquidar, or vehicle. Samples were incubated for 20 minutes at 37°C, then resuspended in prewarmed culture medium and incubated for a further 10 minutes to ensure optimal retention. Calcein AM accumulation was assessed by flow cytometry. ABCB1 expression was assessed using CD243-PE or CD243-APC (clone UIC2, eBioscience), the latter being used when cells were treated with daunorubicin, which has similar excitation and emission spectra to PE.

### ChIP and next-generation sequencing.

Chromatin immunoprecipitation (ChIP) was performed using anti-H3K27Ac (ab4729) and anti-H3K9me3 (ab8898, Abcam). 10^8^ cells were used for each precipitation using the method described by Lee et al. (51). Briefly, cells were cross-linked with 1% formaldehyde for 10 minutes at room temperature before the reaction was quenched with 0.125 M glycine. Cell pellets were washed twice with PBS and nuclear lysates sonicated for 6 cycles using a Bioruptor Pico (Diagenode). Antibody (10 μg) bound to 100 μL of magnetic beads (Dynabeads Protein G, Invitrogen) was added to each sample and immunoprecipitation performed overnight on a rotator at 4°C and 20 rpm. After 5 washes with RIPA buffer (50 mM HEPES [pH 7.6], 1 mM EDTA, 0.7% Na deoxycholate, 1% NP-40, 0.5 M LiCl), ChIP-bound fractions were extracted by incubation for 15 minutes at 65°C with elution buffer (50 mM Tris-HCl [pH 8], 10 mM EDTA, 1% SDS). Cross-linking was then reversed by incubation at 65°C for 6 hours. RNase A (1 mg/mL) and proteinase K (20 mg/mL) were added to eliminate RNA and protein from the samples. DNA was extracted using phenol/chloroform/isoamyl alcohol and precipitated by addition of 2 volumes of ice-cold 100% ethanol, glycogen (20 μg/μL), 200 mM NaCl and freezing at –80°C for at least 1 hour. Pellets were washed with 70% ethanol and eluted in 50 μL 10 mM Tris-HCl (pH 8.0).

Libraries were prepared for sequencing using a Microplex Library Preparation Kit (Diagenode). Fragments of 200–800 bp were selected using AMPure beads (Beckman Coulter) and quantified by quantitative PCR with a KAPA Library Quantification Kit (Kapa Biosystems). Sequencing was performed using a NextSeq desktop sequencing system (Illumina) with 75-bp, paired-end high output generating 40–65 million reads per sample. Reads were aligned to the human genome (hg38) using BWA-MEM version 0.7.15 (52). Read duplicates were removed using Picard version 2.1.0. Reads were further filtered using Bedtools version 2.25.0 to keep only paired reads that mapped to standard chromosomes and to remove reads with a mapping quality of less than 10. Reads mapped to blacklisted regions defined by ENCODE were then removed using Bedtools (http://mitra.stanford.edu/kundaje). To define H3K9 trimethylation caused by dCas9-KRAB, we subtracted nontargeting control reads from each sgRNA track using the BAMcompare function from deepTools2 (53). Results were correlated with ChIP-Seq from ENCODE (Supplemental Table 3) and publicly available DNase I–hypersensitivity site (DHS) data (14, 23). Motif analysis was performed using JASPAR (http://jaspar.genereg.net). Raw data files for ChIP sequencing are available at the Gene Expression Omnibus with the accession number GSE131825.

### ChIP PCR.

ChIP was performed using anti-H3K27Ac (ab4729, Abcam), anti-ATF4 (ab23760, Abcam), anti-ATF3 (D2Y5W, Cell Signaling Technology), anti–c-JUN (60A8, Cell Signaling Technology), anti-JUND (D17G2, Cell Signaling Technology), and anti-CEBPB (ab322588, Abcam). Cells were cross-linked using ChIP Cross-link Gold (C01019027; Diagenode) for 30 minutes in PBS with 1 mM MgCl_2_ and then with 1% formaldehyde for 10 minutes. The reaction was then quenched with 0.125 M glycine. Cell pellets were washed twice with cold PBS containing protease inhibitors (Complete EDTA-free tablets, Roche). Ten million cells were used per ChIP, as described in the protocol reported by Lee et al. (51). Nuclear lysates were sonicated using a Bioruptor Pico (Diagenode) for either 10 (K562) or 8 (BB953 and BB946) cycles. Immunoprecipitation was performed overnight at 20 rpm and 4°C, with 10 μL magnetic beads (Dynabeads Protein G, Invitrogen) per 1 μg antibody. Washing and DNA extraction were performed as for ChIP sequencing. For ChIP, quantitative PCR assays were performed in 384-well MicroAmp optical reaction plates using TaqMan Fast Universal PCR Mastermix (Life Technologies), and with probes and primers designed using the Universal Probe Library System (Roche). Signal was detected using an ABI PRISM 7900HT Sequence Detection System (Life Technologies). For primer sequences and associated probes, see Supplemental Table 6.

### 4C sequencing.

4C primer sequences and enzyme combinations were selected using the University of Chicago online tool (http://mnlab.uchicago.edu/4Cpd) with coordinates from the ABCB1 promoter active in K562_R1–3 cells (hg38, chr7:87,598,302–87,601,399). 4C sequencing was performed according to the protocol developed by Splinter et al. (54). Briefly, 10^7^ cells were cross-linked with 2% formaldehyde for 10 minutes at room temperature before the reaction was quenched with 0.125 M glycine. Cells were lysed with buffer containing 50 mM Tris-HCl (pH 7.5), 150 mM NaCl, 5 mM EDTA, 0.5% NP-40, 1% TX-100, and 1× complete protease inhibitors (11245200, Roche). The cross-linked nuclear preparation was then incubated with DpnII. Digestion was confirmed by reversing cross-linking for an aliquot and running it on a 0.6% agarose gel. Samples were then ligated overnight at 16°C using T4 DNA ligase (799009, Roche). Ligation efficiency was again confirmed with 0.6% agarose gel. Cross-linking was reversed and DNA extracted using phenol-chloroform; samples were then subjected to a second digestion using Csp6I. Ligation was again performed overnight at 16°C using T4 DNA ligase; DNA was then extracted using phenol-chloroform and purified with a QIAquick PCR purification kit (28104, Qiagen). PCR primers were designed to incorporate 4C primers with a barcode and Illumina adapter sequences: reading primer, 5′ P5-Barcode-Primer 3′; nonreading primer, 5′ P7-Primer 3′; reading, GAGATACCAGGTCTGATC; nonreading, AGGGTAGGTATTCCACTTTT; barcode, CTTGTA; illumina adapter sequence P5, AATGATACGGCGACCACCGAGATCTACACTCTTTCCCTACACGACGCTCTTCCGATCT; P7, CAAGCAGAAGACGGCATACGAGAT; nonreading primer, CAAGCAGAAGACGGCATACGAGATAGGGTAGGTATTCCACTTTT; and reading primer, AATGATACGGCGACCACCGAGATCTACACTCTTTCCCTACACGACGCTCTTCCGATCTCTTGTAGAGATACCAGGTCTGATC.

PCR was performed with Expand Long Template Polymerase (11759060001, Roche) using 3.2 μg of 4C template product, and then purified using a High Pure PCR Product Purification Kit (11732676001, Roche). Library quality was assessed using a 2100 Bioanalyzer (Agilent Technologies). Samples were sequenced with 10% phiX using a MiSeq desktop sequencing system (Illumina) with 75-bp, single-end settings generating a mean of 1.3 million reads per sample. Sequencing data were deconvoluted using cutadapt version 1.18. Reads were mapped and analysis performed using 4Cseqpipe (55).

### CRISPR-dCas9-KRAB enhancer silencing.

CRISPR guides were designed with Off-Spotter (https://cm.jefferson.edu/Off-Spotter/), using putative enhancer sequences from K562_R1–3 H3K27Ac ChIP-Seq data. Several guides were selected for each enhancer and the promoter to allow preliminary screening of sgRNA activity. Guides were chosen to provide relatively even coverage across each enhancer and targeting of both DNA strands (Supplemental Figure 3, A–D). Primers incorporating the sgRNA sequence were designed as follows (primer sequences are shown in Supplemental Table 7; 4 nucleotides [green] were added to the guide sequence to permit ligation to the cut vector and if the guide sequence did not start with a guanine then one was added [yellow] to allow efficient transcription by the U6 promoter):
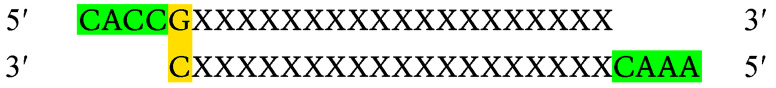


Primers were annealed by heating reagents A (Supplemental Table 8) to 98°C for 5 minutes, then allowing slow cooling by removing the heat block from the heating element until equilibrated to room temperature. Annealed primers were then ligated into pLKO5.sgRNA.EFS.tRFP657 (57824, Addgene) using combined digestion-ligation with *BsmBI* and T4 DNA Ligase (M180A, Promega). Reagents B (Supplemental Table 8) were heated to 55°C for 2 hours; reagents C (Supplemental Table 8) were then added and the temperature reduced to 37°C for 1 hour. Lentivirus was produced using 293FT packaging cells (Life Technologies) cultured in DMEM (Sigma-Aldrich) with 10% FBS. Four micrograms of vector was added to 1 mL DMEM with 21 μL polyethylenimine (Polysciences), 2 μg pCMVd8.91, and 1 μg pMD2.G. The mixture was incubated for 30 minutes at room temperature, then added dropwise to a 10-cm dish containing 75% confluent 293FT cells; medium was replaced after 24 hours. Conditioned medium containing lentivirus was collected at 48 and 72 hours after transfection; packaging cells were removed using a 0.45-μm filter. K562_R1–3 were reselected for 7 days with 500 nM daunorubicin to ensure high-level ABCB1 expression prior to lentiviral transduction with pHR-SFFV-dCas9-BFP-KRAB (46911, Addgene). Transduction was performed by resuspension of 2 × 10^6^ K562 cells in fresh viral supernatant containing 8 μg/mL Polybrene. After 24 hours the medium was exchanged to remove the virus. Seven days later a second transduction was performed using lentivirus containing ligated pLKO5.sgRNA.EFS.tRFP657. After 5 days, expression of mTagBFP, tRFP657, and ABCB1 and calcein AM retention were assessed by flow cytometry. All sgRNAs were screened for activity using K562_R3 (Supplemental Figure 3, E and F). The most active guide for each enhancer was then used to transduce dCas9-KRAB^+^ K562_R1–3. Flow cytometry, RNA extraction, and ChIP were then performed on days 5, 7, and 10 after transduction, respectively. A further assessment of mTagBFP, tRFP657, and ABCB1 expression was made on day 13 to confirm stable expression (Figure 3B).

### Assessment of ABCB1 ATPase activity.

The Pgp-Glo Assay System (V3601, Promega) was used to assess the ability of tosedostat to induce ABCB1 ATPase activity. The assay was performed as described in the product literature. Briefly, Na_3_VO_4_ (0.1 mM), verapamil (0.2 mM), or tosedostat (0.2 mM) was incubated for 40 minutes at 37°C with 5 mM ATP and membranes containing recombinant ABCB1. Residual ATP was then assessed by addition of Ultra-Glo luciferase and incubation at room temperature for 20 minutes. Luminescence was quantified using a GloMax-Multi detection system (Promega). Na_3_VO_4_ inhibits ABCB1 ATPase activity, providing a negative control. Verapamil is a known ABCB1 substrate, inducing ATPase activity and providing a positive control.

### Statistics.

For flow cytometry, quantitative PCR, ChIP PCR, and luciferase assays, statistical significance was determined using the unpaired, 2-tailed Student’s *t* test when comparing 2 experimental groups, or with 1-way ANOVA with Tukey’s correction when comparing 3 or more groups. All tests were performed in Prism 8 (GraphPad). *P* values less than 0.05 were considered statistically significant. The statistical methods used to analyze next-generation sequencing data are detailed in the relevant sections of Methods.

### Study approval.

Primary human AML samples were from the Manchester Cancer Research Centre Tissue Biobank (approved by the South Manchester Research Ethics Committee). Their use was authorized by the Tissue Biobank’s scientific subcommittee, with the informed consent of donors.

## Author contributions

MSW and TCPS designed the study, and MSW and FS performed experiments. MSW, FMRA, and TCPS performed bioinformatics analyses. MSW and TCPS wrote the manuscript. All authors read and approved the final version of the manuscript.

## Supplementary Material

Supplemental data

## Figures and Tables

**Table 2:**
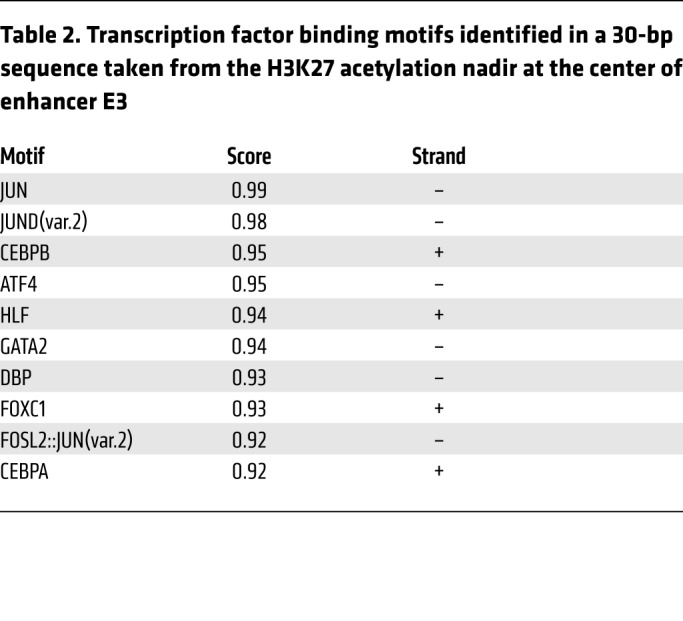
Transcription factor binding motifs identified in a 30-bp sequence taken from the H3K27 acetylation nadir at the center of enhancer E3

**Table 1:**
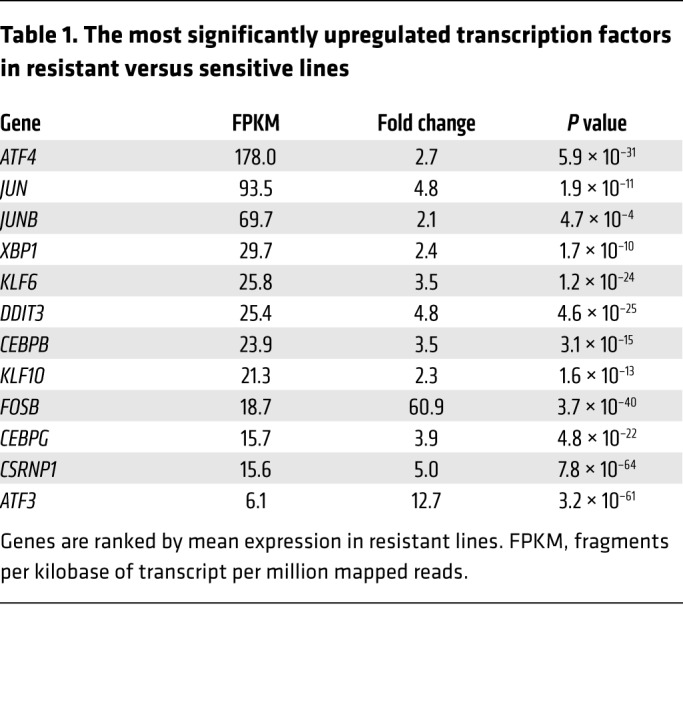
The most significantly upregulated transcription factors in resistant versus sensitive lines

## Version 1 11/26/2019

In-Press Preview

## Version 2 02/04/2020

Electronic publication

## Version 3 03/02/2020

Print issue publication
