# Supplementary material for: A stress-responsive enhancer induces dynamic drug resistance in acute myeloid leukemia
Source: J Clin Invest. 2020 Feb 4;130(3):1217–32. doi: 10.1172/JCI130809 (PMC7269587; doi:10.1172/JCI130809)
Supplement: Supplemental data [file jci-130-130809-s136.pdf]

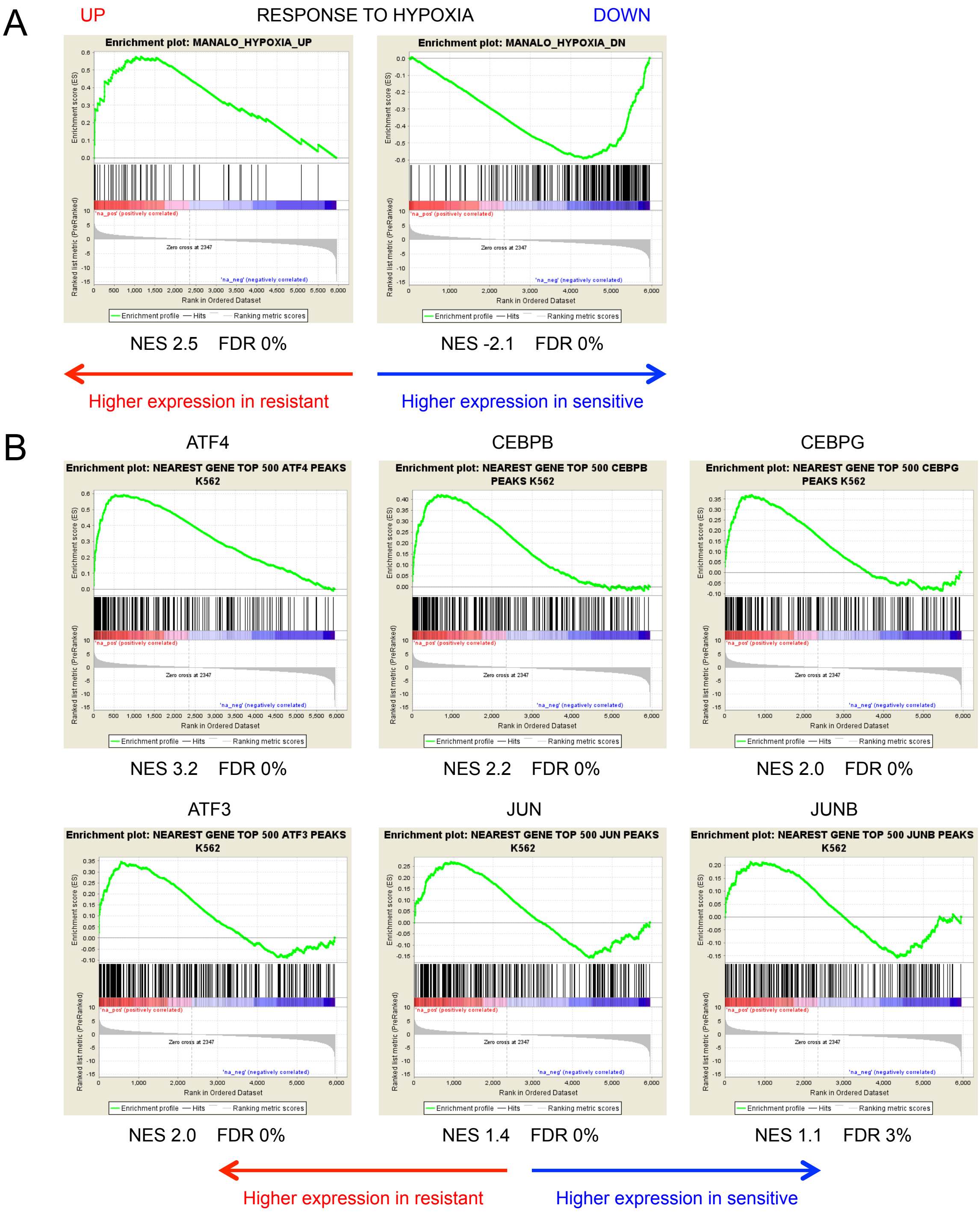

Figure S1. Gene set enrichment analysis in daunorubicin-resistant versus sensitive K562 cells. Related to Figure 2.

(A) GSEA plots show enrichment of a hypoxia gene signature (12) in resistant versus sensitive K562 cells. (B) GSEA plots show enrichment of the top 500 target genes for the indicated transcription factors in resistant versus sensitive K562 cells. The GSEA plot for ATF4 is the same as shown in Figure 2E and is included for reference.

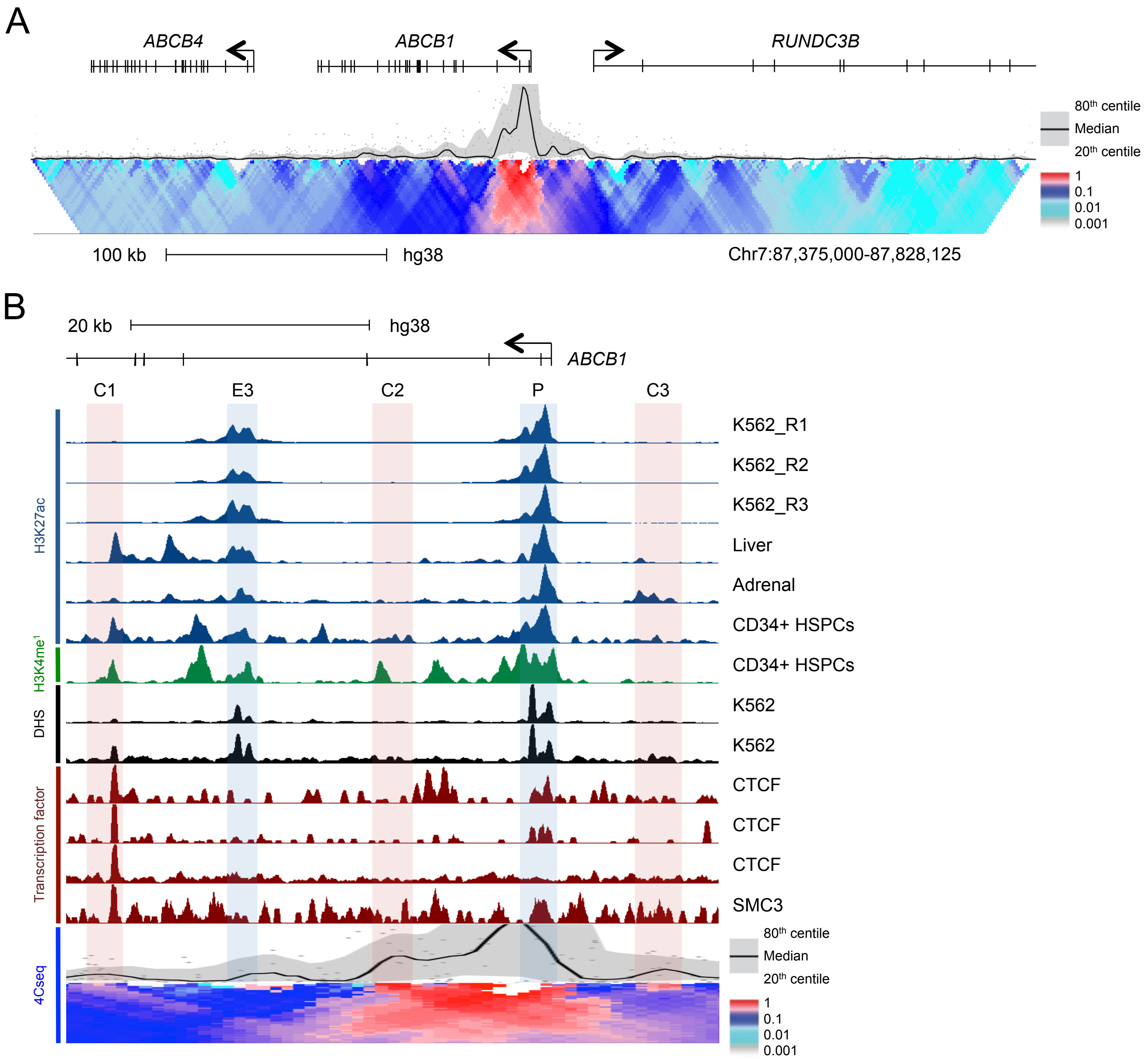

Figure S2. Structure of the *ABCB1* locus. Related to Figure 3.

(A) Contact profile for the region containing *ABCB1* (Chr7: 87,375,000-87,828,125) generated from 4C sequencing of K562_R1 using a viewpoint centred on the *ABCB1* promoter. (B) ChIPseq tracks for H3K27ac, H3K4Me[1], CTCF and SMC3 in K562 cells (ENCODE); and DNAse-seq (ENCODE). Regions of contact that do not contain an active enhancer in K562_R1-3 are highlighted in red (C1-3). H3K27ac ChIPseq tracks and 4Cseq contact profile from K562_R1-3 are included for reference.

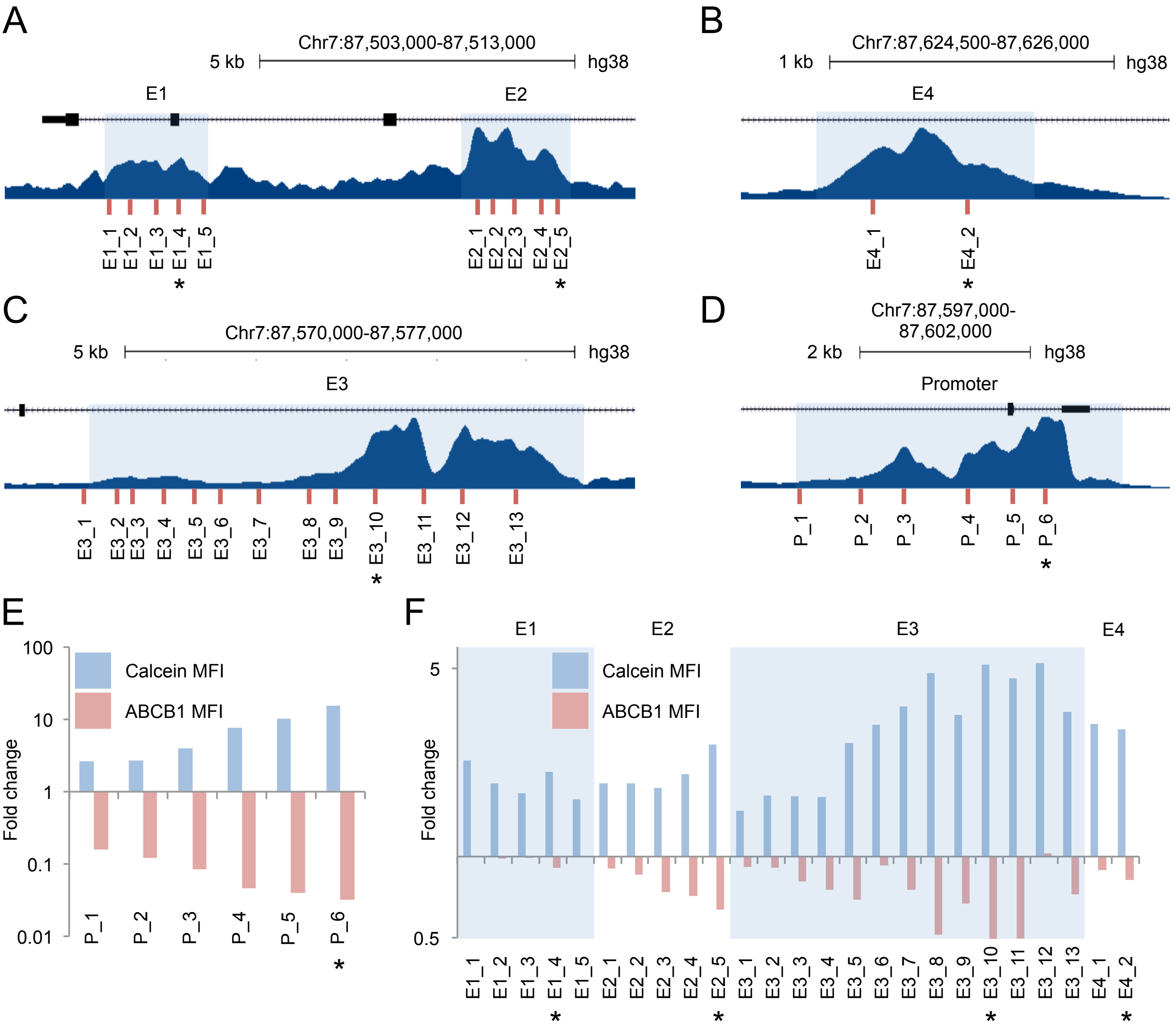

Figure S3. Screening of sgRNAs for promoter and enhancer silencing. Related to Figure 3.

(A-D) ChIPseq tracks for H3K27ac in resistant K562 show the position of the sgRNA sequences designed to target the promoter (D) and each enhancer (A-C). (E) Bar chart shows the change in ABCB1 expression and calcein AM retention following dual infection of K562_R3 with pHR-SFFV-dCas9-BFP-KRAB and pLKO5.sgRNA.EFS.tRFP657 containing the indicated promoter-targeting sgRNA. (F) As for (E), but shows data for each enhancer-targeting sgRNA. *indicates the sgRNA chosen for the promoter and each enhancer.

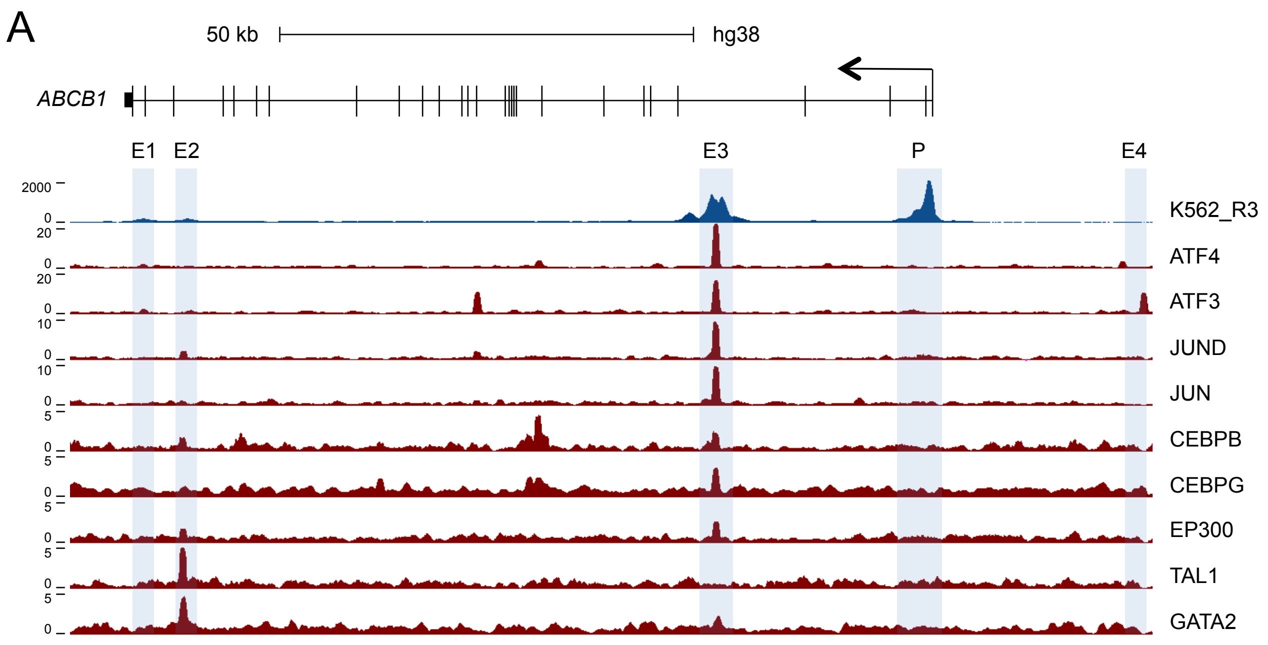

Figure S4. Transcription factor binding within *ABCB1*. Related to Figure 4.

(A) ChIPseq tracks for the indicated transcription factors in unmanipulated K562 (ENCODE) within the *ABCB1* gene. H3K27ac ChIPseq tracks from K562_R3 are included for reference. The promoter (P) and enhancers (E1-4) are highlighted in blue.

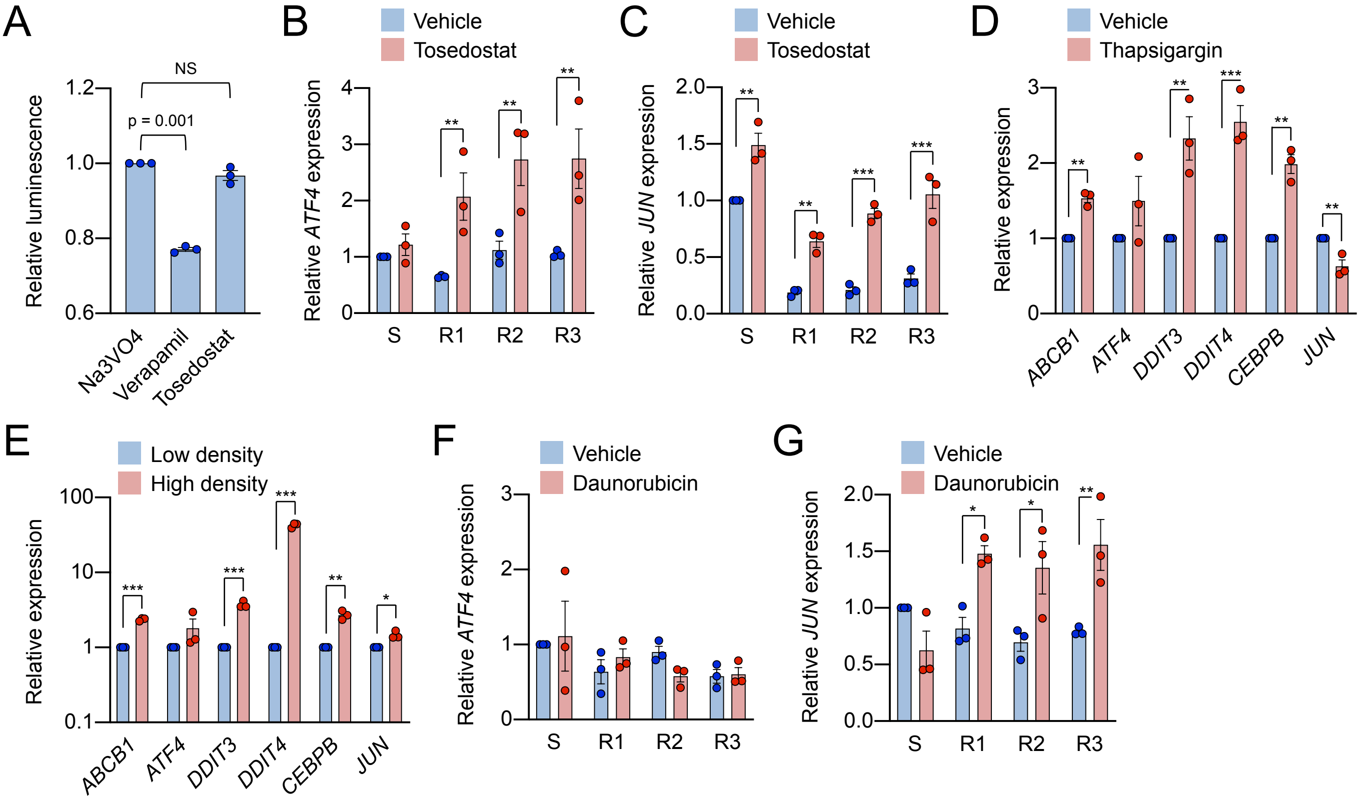

Figure S5. Stress induces expression of *ABCB1*. Related to Figure 5.

(A) Mean±SEM relative luminescence from luciferase assay of ABCB1 ATPase activity following treatment with Na_3VO_4 (an ABCB1 inhibitor), verapamil (a known ABCB1 substrate) or tosedostat (n=3). NS, not significant by one way ANOVA with Tukey post hoc test. (B-C) Mean±SEM expression of (B) *ATF4* and (C) *JUN* by quantitative PCR relative to unmanipulated drug sensitive K562 cells following exposure to tosedostat 50μM for 48hrs (n=3). **p<0.01, ***p<0.001 by unpaired t-test. (D) Mean±SEM expression of the indicated genes by quantitative PCR relative to vehicle for unmanipulated drug sensitive K562 cells following exposure to thapsigargin 100nM for 48hr (n=3). **p<0.01, ***p<0.001 by unpaired t-test. (E) Mean±SEM expression of the indicated genes by quantitative PCR relative to drug sensitive K562 cells cultured at low density (<$5x10^5$ cells/ml) following 48hr of high density culture (>10^6 cells/ml, n=3). *p<0.05, **p<0.01, ***p<0.001 by unpaired t-test. (F-G) Mean±SEM expression of (F) *ATF4* or (G) *JUN* by quantitative PCR relative to unmanipulated drug sensitive K562 cells following exposure to daunorubicin 100nM (S) or 500nM (R1-3) for 48hrs (n=3). *p<0.05, **p<0.01 by unpaired t-test.

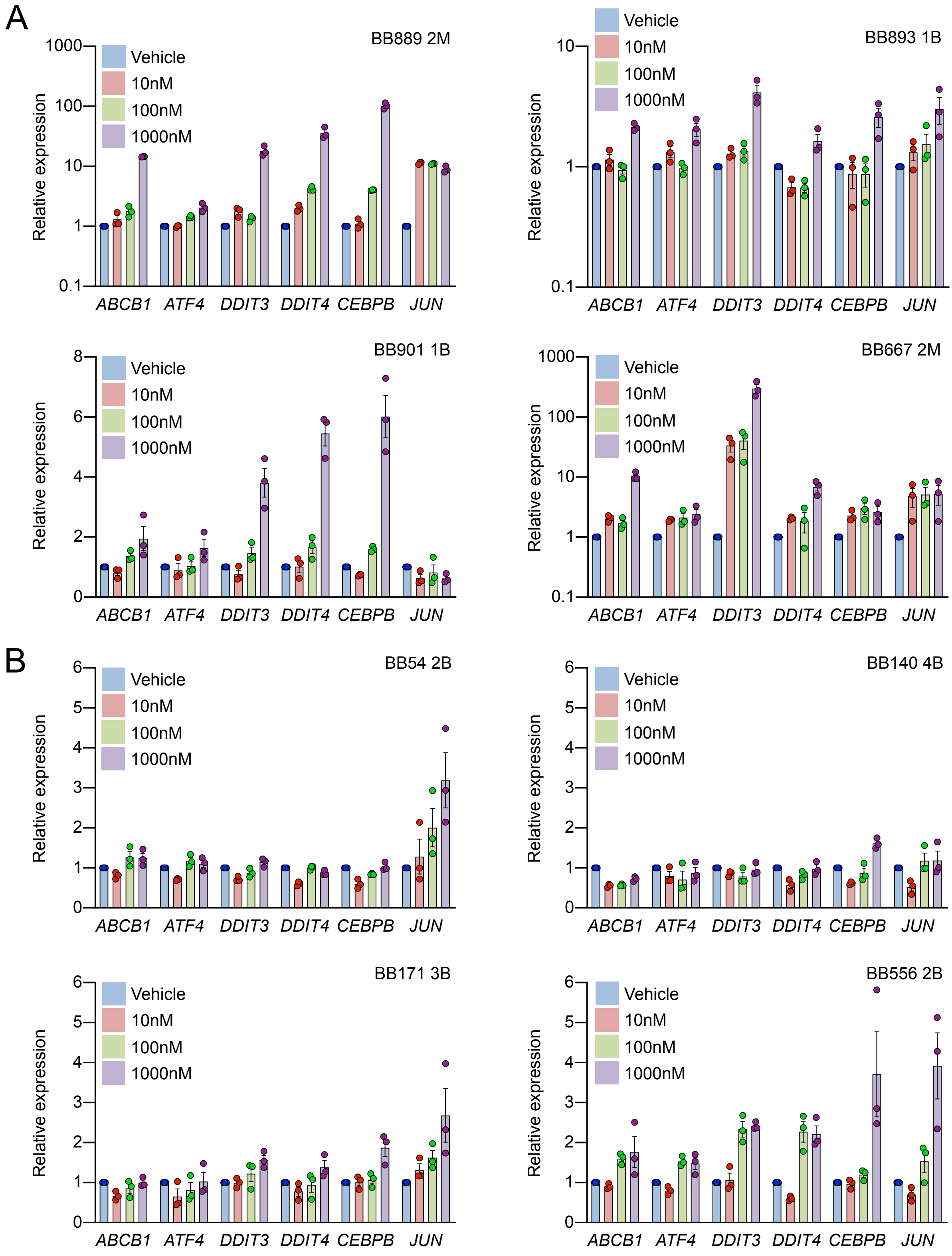

Figure S6. Fresh primary AML blasts respond to acute daunorubicin exposure with *ABCB1* induction. Related to Figure 6.

(A-B) Mean±SEM relative expression of the indicated genes following exposure of fresh (A) or freeze-thawed (B) primary AML blast cells to the indicated doses of daunorubicin for 18 hours (n=3). BB numbers indicate Biobank identifier.

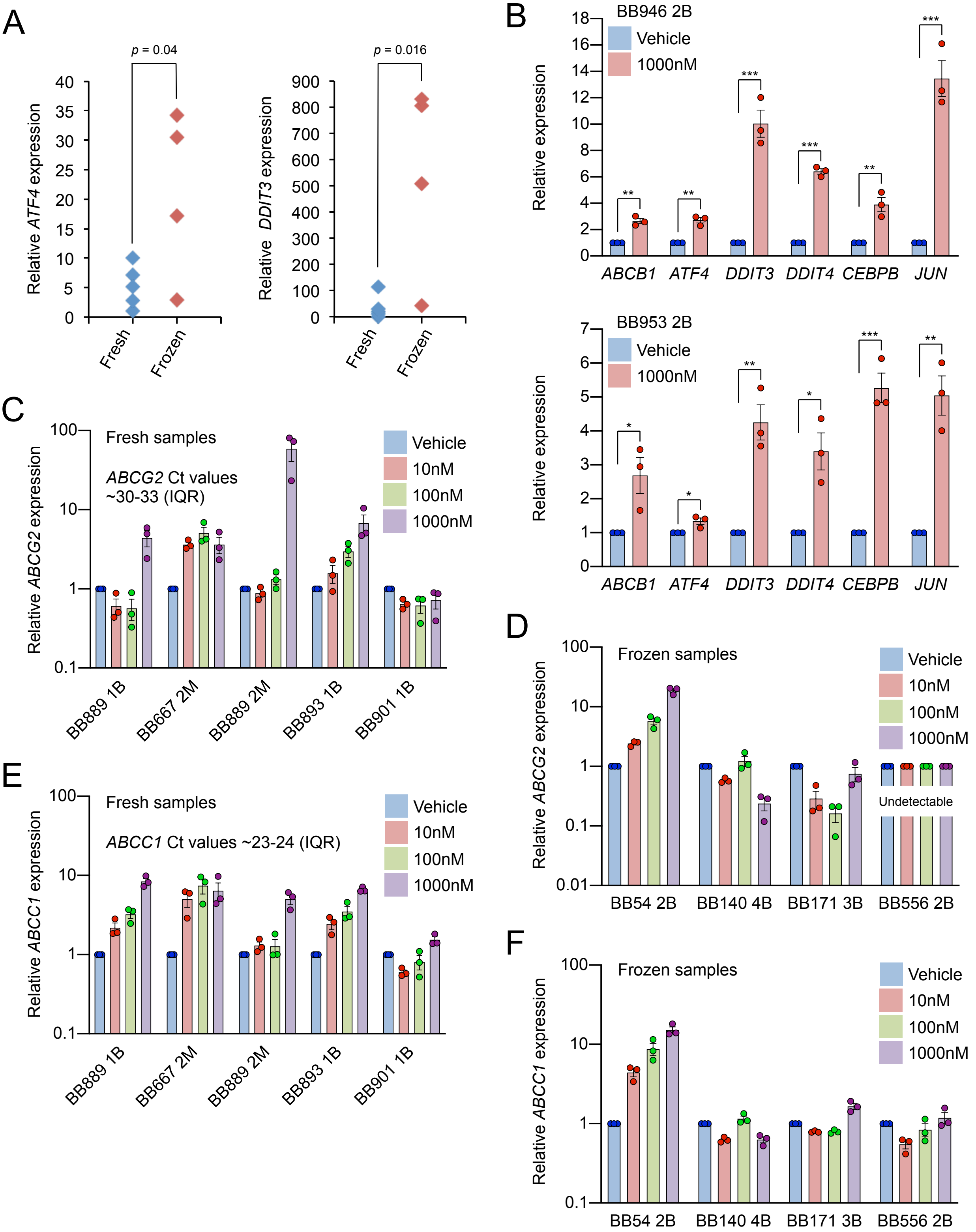

Figure S7. Fresh primary AML blasts respond to acute daunorubicin exposure with *ABCB1* induction. Related to Figure 6.

(A) Expression of *ATF4* and *DDIT3* relative to BB667 for all fresh and frozen primary AML samples. Fresh were compared with frozen using an unpaired t-test. (B) Mean±SEM relative expression of the indicated genes following exposure of fresh primary AML blast cells (BB946 & BB953) to 1000nM daunorubicin for 18 hours (n=3). *p<0.05, **p<0.01, ***p<0.001 by unpaired t-test. (C-D) Mean±SEM relative expression of *ABCG2* following exposure of (C) fresh or (D) freeze-thawed AML blast cells to the indicated doses of daunorubicin for 18 hours (n=3). (E-F) Mean±SEM relative expression of *ABCC1* following exposure of (E) fresh or (F) freeze-thawed AML blast cells to the indicated doses of daunorubicin for 18 hours (n=3). BB numbers indicate Biobank identifier. IQR, interquartile range.

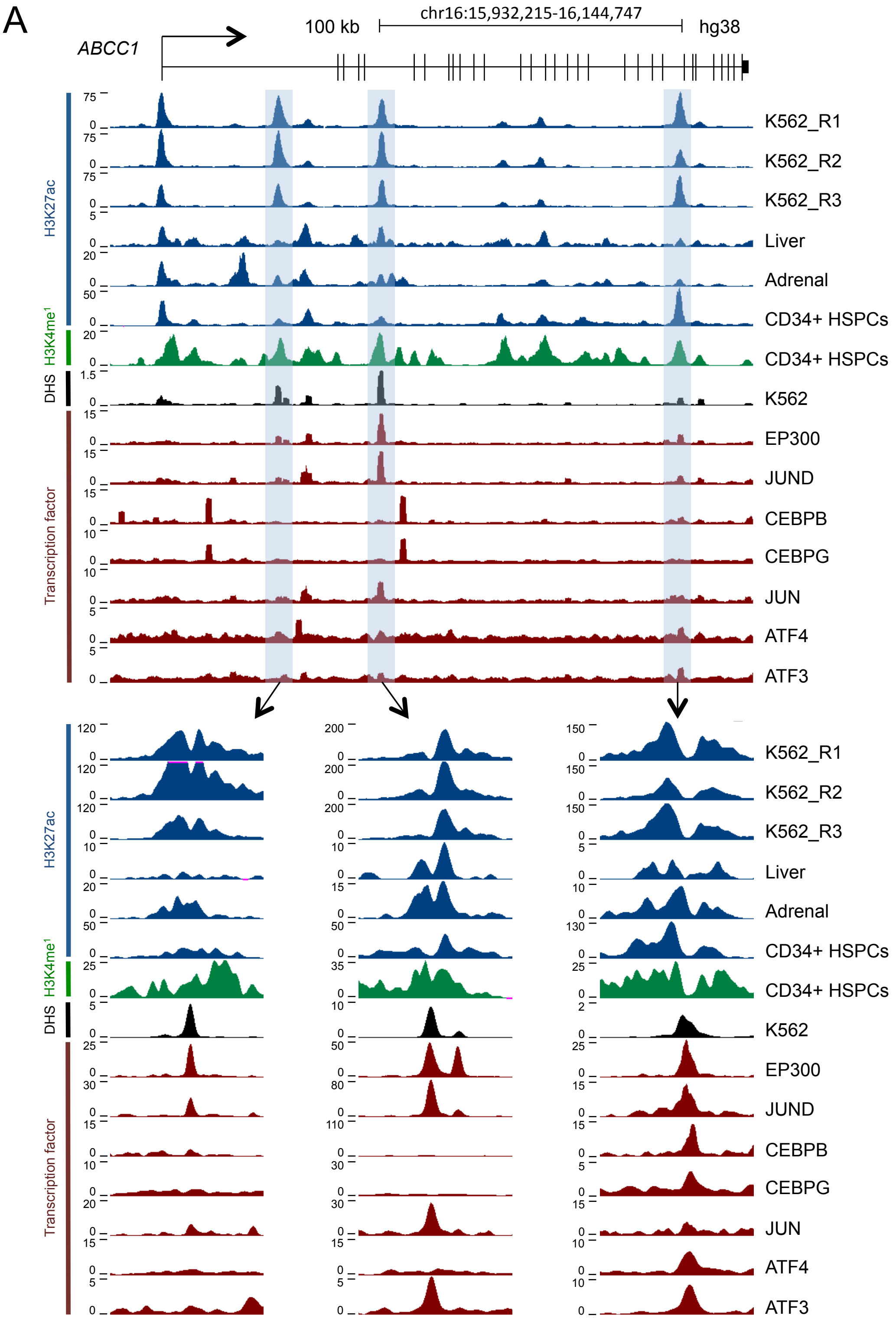

Figure S8. Regulatory element landscape of *ABCC1*. Related to Figure 6.

(A) ChIPseq tracks for H3K27Ac, H3K4Me1 and DNAse-seq surrounding *ABCC1* (chr16:15,932,215-16,144,747; hg38) in the indicated human cells and tissues, including CD34^{+} hematopoietic stem and progenitor cells (ENCODE) and K562_R1-3 (our data). Putative enhancers are highlighted in blue. ChIPseq tracks for the indicated transcription factors are for unmanipulated K562 cells from ENCODE (14).

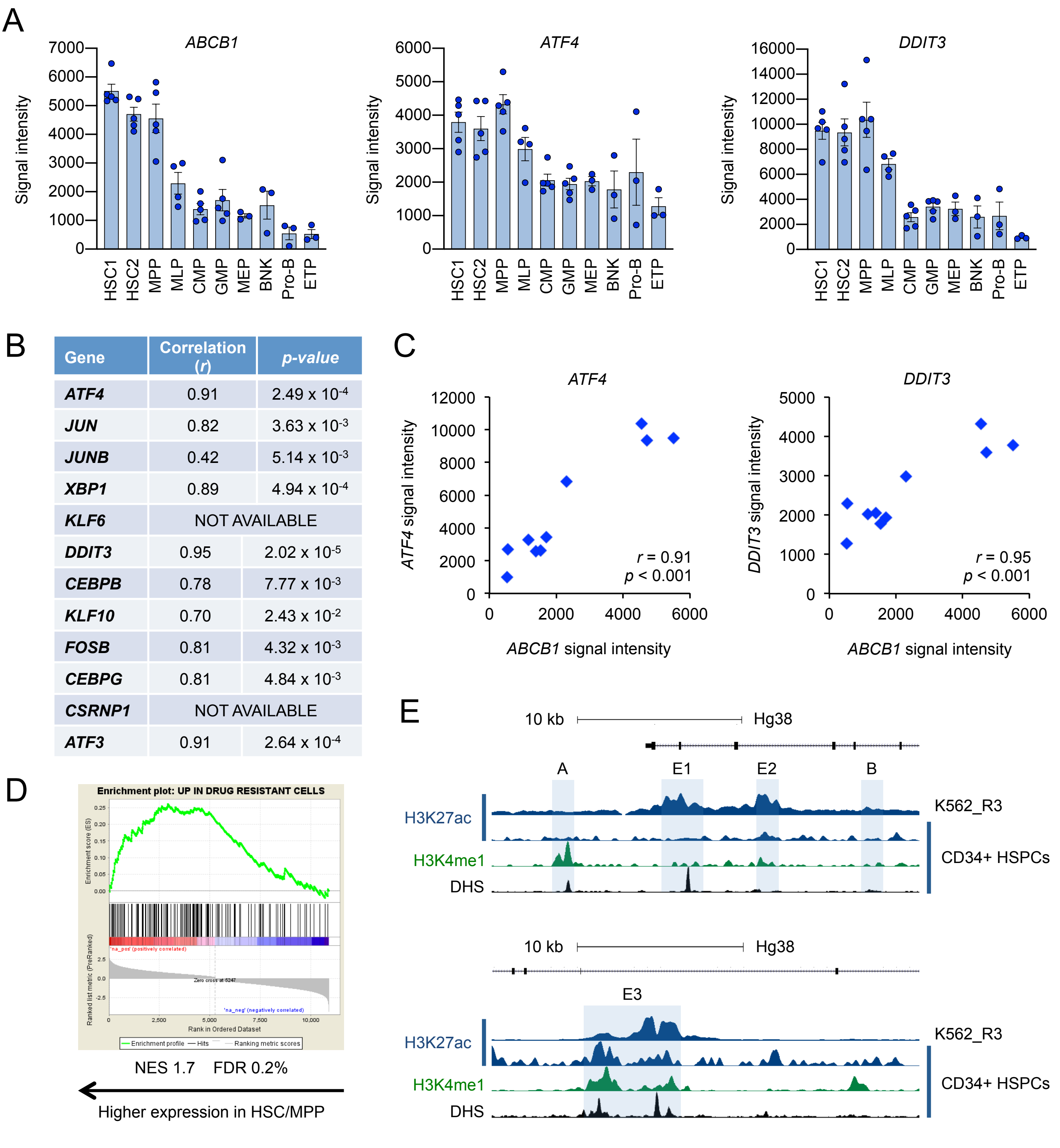

Gene	Correlation (r)	p-value
ATF4	0.91	2.49×10^{-4}
JUN	0.82	3.63×10^{-3}
JUNB	0.42	5.14×10^{-3}
XBP1	0.89	4.94×10^{-4}
KLF6	NOT AVAILABLE	
DDIT3	0.95	2.02×10^{-5}
CEBPB	0.78	7.77×10^{-3}
KLF10	0.70	2.43×10^{-2}
FOSB	0.81	4.32×10^{-3}
CEBPG	0.81	4.84×10^{-3}
CSRNP1	NOT AVAILABLE	
ATF3	0.91	2.64×10^{-4}

Figure S9. Adaptive stress signalling and *ABCB1* expression across normal hematopoiesis. Related to Figure 6.

(A) Expression of *ABCB1*, *ATF4* and *DDIT3* in sorted cord blood populations (26). Data are shown as mean normalised signal intensity ±SEM (n=3-5). (B) Table shows transcription factors upregulated in K562_R1-3 (Figure 2E), their correlation with *ABCB1* across normal hematopoiesis and associated p-value. *r* represents the Pearson product-moment correlation coefficient. (C) Scatter plots show *ATF4* or *DDIT3* expression versus *ABCB1* expression in sorted cord blood populations (26). *r* represents the Pearson product-moment correlation coefficient. (D) GSEA plot shows the enrichment of genes upregulated in K562_R1-3 (Figure 2C, Table S2) in a list ranked by fold change expression between HSC/MPP and myeloid progenitor cells (CMP, GMP, MEP) (26). (E) ChIPseq tracks for H3K27Ac, H3K4me1 and DNAse-seq (ENCODE) in the region of E1/E2 (top) and E3 (bottom) from normal CD34^{+} HSPCs. H3K27Ac ChIPseq tracks from K562_R3 are included for reference.

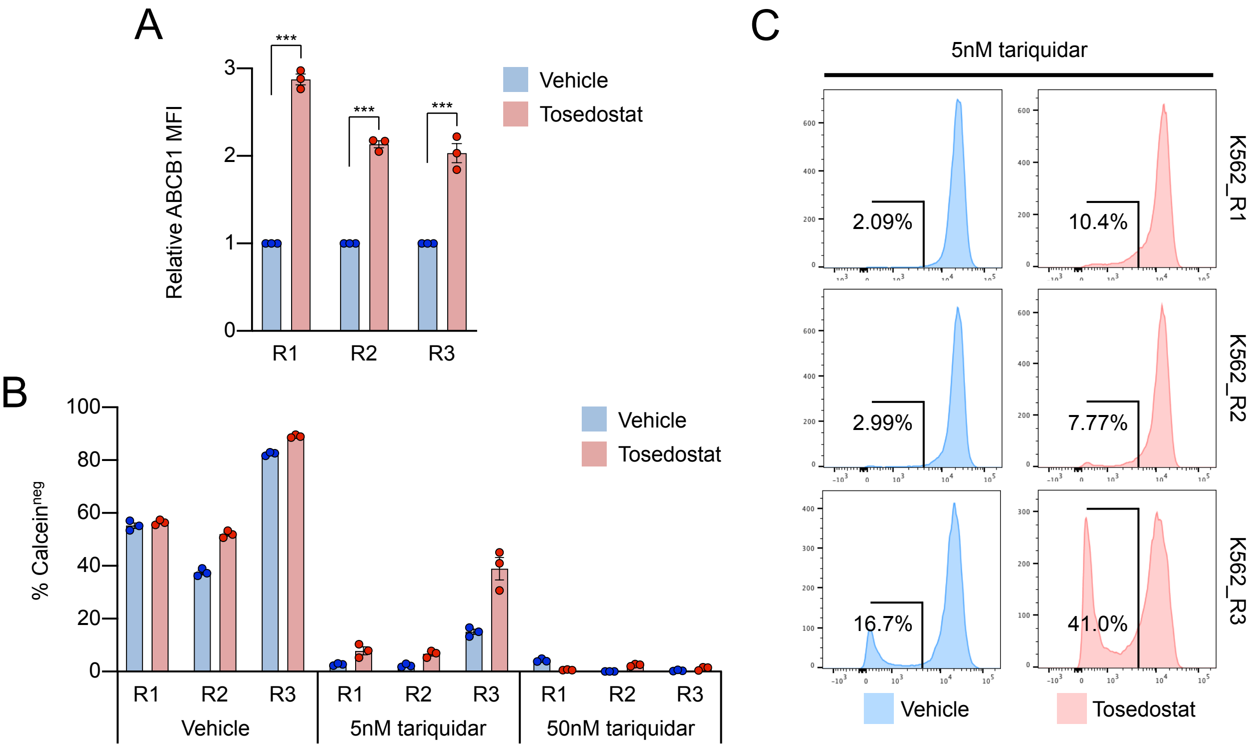

Figure S10. Activation of an ISR-like response facilitates escape from ABCB1 inhibition. Related to Figure 7.

(A) Mean±SEM relative ABCB1 MFI in K562_R1-3 following exposure to 50μM tosedostat or vehicle for 48hrs (n=3). ***p<0.001 by unpaired t-test. (B) Proportion of cells that are calcein AM negative following exposure of K562_R1-3 to the indicated conditions as determined by flow cytometry (n=3). (C) As for (B), but showing individual flow histograms for each of the indicated conditions.

Table S3. ENCODE data used for analyses. Related to Figures 3, 4, 6, S2, S4, S8 & S9.

Upper table shows ChIPseq data from ENCODE used to assess transcription factor (TF) binding to enhancers in K562 cells (14). The availability of data for all of the TFs upregulated in resistant K562s (Figure 2E) is shown. *peak not contained within region of enhancer acetylation but within 1 kb. Lower table shows additional ENCODE data used to define *ABCB1* locus structure and enhancer usage in ABCB1 expressing tissues (14).

TF	Cell type	Lab	Experiment number	Evidence of TF binding				
				E1	E2	E3	Promoter	E4
**ATF4**	K562	Michael Snyder, Stanford	ENCSR145TSJ	Wk		YES		YES*
**JUN**	K562	Michael Snyder, Stanford	ENCSR000EFS			YES		
**JUNB**	K562	Richard Myers, HAIB	ENCSR795IYP			YES		
**XBP1**	Unavailable							
**KLF6**	Unavailable							
**DDIT3**	Unavailable							
**CEBPB**	K562	Michael Snyder, Stanford	ENCSR000EHE		YES	YES		
**KLF10**	Unavailable							
**FOSB**	Unavailable							
**CEBPG**	K562	Michael Snyder, Stanford	ENCSR490LWA			YES		
**CSRNP1**	Unavailable							
**ATF3**	K562	Michael Snyder, Stanford	ENCSR028UIU	Wk		YES		YES*
**TAL1**	K562	Michael Snyder, Stanford	ENCSR000EHB		YES			
**GATA2**	K562	Kevin White, UChicago	ENCSR000DKA		YES	YES		
**JUND**	K562	Michael Snyder, Stanford	ENCSR000EGN		YES	YES		
**EP300**	K562	Michael Snyder, Stanford	ENCSR000EGE		YES	YES		

Track	Cell type	Lab	Experiment number
**CTCF**	K562	Bradley Bernstein, Broad	ENCSR000AKO
**CTCF**	K562	Michael Snyder, Stanford	ENCSR000EGM
**CTCF**	K562	Richard Myers, HAIB	ENCSR000BPJ
**SMC3**	K562	Michael Snyder, Stanford	ENCSR000EGW
**DNase-seq**	K562	John Stamatoyannopoulos, UW	ENCSR000EOY
**DNase-seq**	K562	John Stamatoyannopoulos, UW	ENCSR000EOT
**H3K27ac**	Adult liver	Bradley Bernstein, Broad	ENCSR230IMS
**H3K27ac**	Adult adrenal gland	Bradley Bernstein, Broad	ENCSR189QAD
**H3K27ac**	CD34+ haematopoietic progenitor	Bradley Bernstein, Broad	ENCSR891KSP
**H3K4me^1**	CD34+ haematopoietic progenitor	Bradley Bernstein, Broad	ENCSR691CSS

Table S4. Primary AML samples. Related to Figures 6, S6 & S7.

Clinical characteristics of AML patient samples used for assessment of *ABCB1* and *ATF4* expression (Figure 6A). Samples used for ChIPseq are highlighted in green. Samples used for daunorubicin exposure experiments are highlighted in red (Figures 6F, S6 & S7). Samples used for ChIP-PCR are highlighted in blue (Figure 6G). *at diagnosis; PB = peripheral blood; BM = bone marrow; sl = stemline; sdl = sideline; der = derivative; t = translocation; del = deletion; add = additional material of unknown origin; idem = denotes the stemline karyotype in a subclone; inv = inversion; cp = composite karyotype.

ID	Sample	Disease Status	Gender	Age*	Blast %	Cytogenetics*
14 T10	PB	Relapse	M	52	100	46,XY [20]
53 T1	PB	Relapse	M	74	97	46,XY [20]
54 T2	PB	Refactory	F	45	99	47,XY,+11[1]/48,sl,+8[7]/49,sdl,+4[2]
140 T4	PB	Relapse	M	22	91	46,XY [20]
148 T7	PB	Relapse	M	72	80	47,XY,+11[1]/48,sl,+8[7]/49, sdl,+4[2]
160 T9	PB	Relapse	F	71	97	46,XX [20]
161 T30	PB	Relapse	M	40	91	46,XY,t(6;11)(q27;q23)[10]/ 48,idem,+der(6)t(6;11),+21[4]
171 T3	PB	Relapse	F	26	89	46,XX,t(9;11)(p22;q23),der(21;22)(q10;q10), +der(21;22)[cp10]
225 T15	PB	Relapse	F	66	96	46,XX [20]
225 T2	PB	Relapse	F	57	97	46,XX [20]
310 T2	PB	Relapse	F	73	85	46,XX [20]
338 T2	PB	Relapse	F	73	89	46,XX [20]
338 T4	PB	Refactory	F	66	86	46,XX [20]
355 T15	BM	Relapse	M	73	92	46,XY[20]
355 T22	PB	Relapse	M	73	95	46,XY[20]
380 T1	PB	Relapse	M	66	92	46,XY [20]
485 T10	PB	Relapse	M	66	87	46,XY [20]
497 T2	PB	Relapse	M	15	97	46,XY,t(8;21)(q22;q22)
546 T6	PB	Relapse	F	77	82	46,XX [20]
556 T1	BM	Relapse	M	47	100	46,XX [20]
556 T2	PB	Relapse	M	47	95	46,XY [20]
572 T11	PB	Relapse	M	60	95	46,XY [20]
727 T1	PB	Relapse	M	37	95	Inv(16)
764 T2	BM	Presentation	F	73	45	Failed
773 T2	PB	Presentation	M	50	22	46,XY [20]
782 T2	PB	Presentation	F	34	92	46,XX,t(9;11)(p21.3;q23)[10]
667 T2	BM	Relapse	F	59	60	del(5q)
893 T1	PB	Relapse	M	72	22	47,XY,+13[7]/49-51,idem,+8,+9,+14,+15[cp10]/46,XY[13]

889 T2	BM	Relapse	M	74	97	46,XY [20]
889 T1	PB	Relapse	M	74	97	46,XY [20]
901 T1	PB	Relapse	M	69	100	46,XY [20]
946 T2	PB	Presentation	M	64	73	46,XY,t(6;9)(p22;q34)
953 T2	PB	Presentation	M	53	>90	46,XY,inv(16)(p13q22)[15]/46,XY[5]

Table S5. UPL primers and probes used for quantitative PCR.

UPL primers and probes used for quantitative PCR			
**Gene**	**Species**	**Primer**	**Probe**
ACTB	Human	F: ATTGGCAATGAGCGGTTC<br>R: GGATGCCACAGGACTCCAT	11
ABCB1	Human	F: GGAAATTTAGAAGATCTGATGTCAAAC<br>R: ACTGTAATAATAGGCATACCTGGTCA	65
ATF4	Human	F: TGGTCAGTCCCTCCAACAAC<br>R: CTATACCCAACAGGGCATCC	88
DDIT3	Human	F: CAGAGCTGGAACCTGAGGAG<br>R: CCATCTCTGCAGTTGGATCA	9
DDIT4	Human	F: CTGGAGAGCTCGGACTGC<br>R: TCCAGGTAAGCCGTGTCTTC	56
CEBPB	Human	F: CGCTTACCTCGGCTACCA<br>R: ACGAGGAGGACGTGGAGAG	74

Table S6. UPL primers and probes used for ChIP PCR.

UPL primers and probes used for ChIP PCR			
**Target**	**Species**	**Primer**	**Probe**
**E3_1***	Human	F: TGGAGGGTCCAGGTAAAAGA<br>R: GGTCTTGCTCTAGGGTCTGC	12
**E3_2***	Human	F: GCCTGACCTTCCTGACACAC<br>R: TGCCAGCACTTGGTCTTTTA	89
**C1**	Human	F: GGGCCTATTAGAGGGTGGAG<br>R: CCAAGTATTAAGCCTAGTACCCATTAG	52
**E3***	Human	F: TGCAATAGAACTCCGTAGTAATTGA<br>R: CAGTTTTCTATGACCTCACACCA	69

*E3_1 and E3_2 were used to assess H3K27 acetylation whilst E3 was used to assess transcription factor binding.

Table S7. Primer sequences used for CRISPR-dCas9-KRAB enhancer silencing.

Name	Primers	Strand
E1_1	F: CACCGCCTAATTTGGGTTAGTTCCG<br>R: AAACCGGAACTAACCCAAATTAGGC	+
E1_2	F: CACCGCTGGCCGGCACCTACTGCCG<br>R: AAACCGGCAGTAGGTGCCGGCCAGC	-
E1_3	F: CACCGAACGCAAACTGATCTAGTGA<br>R: AAACTCACTAGATCAGTTTGCGTTC	+
E1_4	F: CACCGAGCACTAAAGTAGGAGACAA<br>R: AAACTTGTCTCCTACTTTAGTGCTC	-
E1_5	F: CACCGTCATCTTACTTGTAACCAGC<br>R: AAACGCTGGTTACAAGTAAGATGAC	+
E2_1	F: CACCGCTTGGAACTAATAAGGTTAT<br>R: AAACATAACCTTATTAGTTCCAAGC	-
E2_2	F: CACCGTCCAGGTTTATACCCCTGGG<br>R: AAACCCCAGGGGTATAAACCTGGAC	+
E2_3	F: CACCGAACTAGGAAAGTAAACCTAT<br>R: AAACATAGGTTTACTTTCCTAGTTC	+
E2_4	F: CACCGGGGGCATTCCCCTTTCAAG<br>R: AAACCTTGAAAGGGGAATGCCCCC	-
E2_5	F: CACCGAGGGAGGCAAGGCTGTAGTC<br>R: AAACGACTACAGCCTTGCCTCCCTC	+
E3_1	F: CACCGACACACTTATTTTTTAACAA<br>R: AAACTTGTTAAAAAATAAGTGTGTC	+
E3_2	F: CACCGTTCCTACCCCTGTCAAAAAC<br>R: AAACGTTTTTGACAGGGGTAGGAAC	-
E3_3	F: CACCGCAGCAATGTATACGATTAAC<br>R: AAACGTTAATCGTATACATTGCTGC	+
E3_4	F: CACCGTTACGGTCTGAATACTTAT<br>R: AAACATAAGTATTCAGACCGTAAC	-
E3_5	F: CACCGAAACAGGACATTGAATAGTC<br>R: AAACGACTATTCAATGTCCTGTTTC	+
E3_6	F: CACCGAAAGTAAAAAATTTTGTTCA<br>R: AAACTGAACAAAATTTTTTACTTTC	+
E3_7	F: CACCGCCTACTTATAAGTGAAAACG<br>R: AAACCGTTTTCACTTATAAGTAGGC	-
E3_8	F: CACCGTGTTTCCCAGAACTCTATGC<br>R: AAACGCATAGAGTTCTGGGAAACAC	+
E3_9	F: CACCGAGAACTAATATGATGTATCA<br>R: AAACTGATACATCATATTAGTTCTC	-
E3_10	F: CACCGTGCTCTAGGGTCTGCTTAGG<br>R: AAACCCTAAGCAGACCCTAGAGCAC	-
E3_11	F: CACCGTCCATTCTGCTTTCTCTAAC<br>R: AAACGTTAGAGAAAGCAGAATGGAC	+
E3_12	F: CACCGATACATTAGACCTTTCTACC<br>R: AAACGGTAGAAAGGTCTAATGTATC	+
E3_13	F: CACCGCAGTTTGGGCCAATGCCATA<br>R: AAACTATGGCATTGGCCCAAACTGC	-
P_1	F: CACCGAATACCTACCCTCCACTCTA<br>R: AAACTAGAGTGGAGGGTAGGTATTC	+
P_2	F: CACCGTAGTAAATGACTCATCACT<br>R: AAACAGTGATGAGTCATTTACTAC	-
P_3	F: CACCGCTGGCTGAAATTAGCTACTG<br>R: AAACCAGTAGCTAATTTCAGCCAGC	+
P_4	F: CACCGGTGAATGACTAAGAACGGT<br>R: AAACACCGTTCTTAGTCATTCACC	+
P_5	F: CACCGATCTTGAAGGGGACCGCAA<br>R: AAACTTGCGGTCCCCTTCAAGATC	-
P_6	F: CACCGGTCCAGTGCCACTACGGTT<br>R: AAACAACCGTAGTGGCACTGGACC	+
E4_1	F: CACCGACATTTGAAAAATAACGTTA<br>R: AAACTAACGTTATTTTTCAAATGTC	+
E4_2	F: CACCGATTACCTCATCATTTAAGA<br>R: AAACTCTTAAATGATGAGGTAATC	+

Table S8. Reagents used for CRISPR guide RNA cloning.

Reagent	Volume (µl)
**A**	
NEB Buffer 2.1	5
Sense oligo 100uM	5
Antisense oligo 100uM	5
ddH20	35
**B**	
RE Buffer (3.1)	2
1µg Vector	0.8
BsmBI	1
ddH20	16.2
**C**	
T4 ligase buffer	2.5
Annealed oligo	1
T4 Ligase	1.5
